# The influence of caging, bedding, and diet on the composition of the microbiota in different regions of the mouse gut

**DOI:** 10.1038/s41598-018-21986-7

**Published:** 2018-03-06

**Authors:** Aaron C. Ericsson, Jonalyn Gagliardi, Delia Bouhan, William G. Spollen, Scott A. Givan, Craig L. Franklin

**Affiliations:** 1 University of Missouri Mutant Mouse Resource and Research Center, Columbia, USA; 2 University of Missouri Metagenomics Center, Columbia, USA; 3 University of Missouri, College of Veterinary Medicine, Department of Veterinary Pathobiology, Columbia, USA; 4 University of Missouri, Informatics Research Core Facility, Columbia, USA

## Abstract

Countless studies have identified differences between the gut microbiota of humans affected with myriad conditions and healthy individuals, and animal models are commonly used to determine whether those differences are causative or correlative. Recently, concerns have arisen regarding the reproducibility of animal models between institutions and across time. To determine the influence of three common husbandry-associated factors that vary between institutions, groups of weanling mice were placed in either static or ventilated microisolator caging, with either aspen or paperchip bedding, and with one of three commonly used rodent chows, in a fully crossed study design. After thirteen weeks, samples were collected from multiple regions of the gastrointestinal tract and characterized using culture-independent sequencing methods. Results demonstrated that seemingly benign husbandry factors can interact to induce profound changes in the composition of the microbiota present in certain regions of the gut, most notably the cecum, and that those changes are muted during colonic transit. These findings indicate that differences in factors such as caging and bedding can interact to modulate the gut microbiota that in turn may affect reproducibility of some animal models, and that cecal samples might be optimal when screening environmental effects on the gut microbiota.

## Introduction

The collection of microorganisms present in the gastrointestinal tract (GIT) of multicellular animals, often generically referred to as the gut microbiota (GM), represents a complex and diverse ecological system with an undeniable influence on host health and disease susceptibility^1,2^. Conversely, the composition of the GM can be affected by the health status of the host, often leading to the question of whether differences observed between healthy and affected individuals in the GM composition are causative or merely correlative^3^. Thus, a comparative medicine approach employing animal (often rodent) models for prospective studies with longitudinal sample collection is vital in this rapidly growing area of research. Despite the common goal of optimal health status and elimination of pathogens, the husbandry of laboratory rodents is not however standardized. In addition to the obvious differences between barrier-housed and conventionally housed rodents, there are many subtle differences within either type of housing including, but not limited to, bedding type^4^; commercial source, formulation, and post-manufacture treatment of food^5^; air flow (static versus ventilated)^6,7^, commercial source, and overall size of caging; and housing density^8^. The influence of these often overlooked variables on the composition of the GM is largely unknown and there is the potential that husbandry-induced changes in the GM could contribute to a lack of reproducibility between labs or institutions^9,10^.

Additionally, the majority of research involving the GM, whether performed in humans or animal models, focuses on the fecal microbiota as the necessary samples can be acquired noninvasively, thus allowing for longitudinal studies with repeated measures. There are however distinct differences between regions of the GIT with regard to overall tissue function, cell type distribution^11^, energy utilization^12,13^, and the density and composition of the luminal microbiota^2,14^. It is thus reasonable to believe that disease or treatment-induced effects on the GM may go undetected in studies based purely on fecal samples. Considering the regional differences in the recognition of, and response to, the gut microbiota via mucosa-associated lymphoid tissue (MALT)^15,16^, any such undetected differences may very well be of physiological relevance. Thus, we hypothesized that differences in husbandry such as bedding and caging type or dietary formula would result in changes in the intestinal microbiota, and that those differences would be more evident in certain regions of the GIT.

To determine the influence of three common husbandry variables (bedding, cage ventilation, and diet) on the composition of the GM of laboratory mice, a fully-crossed longitudinal study design was devised, incorporating two different bedding types (compressed paper and aspen chips), two different caging types (static microisolators and individually ventilated caging), and three different dietary formulations purchased from the same source (Purina LabDiet 5008, 5053, and 5058). These are all commonly used research rodent diets, the primary differences being irradiation of LabDiet 5008, and macromolecular content. Specifically, 5008, 5053, and 5058 contain crude protein of at least 23%, 20%, and 20%, and crude fat of at least 6.5%, 4.5%, and 9%, respectively. Fecal samples were collected from 144 mice (*n* = 12/group) one week after arrival at our facility and again 12 weeks later. Moreover, to determine whether any changes detected in the composition of the fecal microbiota following 12 weeks under the different conditions were representative of the microbiota present in other regions of the GIT, samples were also collected at necropsy of the luminal contents of the jejunum, ileum, and cecum. Microbial communities were characterized via 16S rRNA amplicon sequencing using the Illumina MiSeq platform and the influence of caging, bedding, and diet on various aspects of the GM were tested using permutational multivariate analysis of variance (PERMANOVA)^17,18^ and three-way ANOVA via a general linear model, as appropriate.

## Results

### Baseline composition of fecal microbiota is uniform

**Figure 1:**
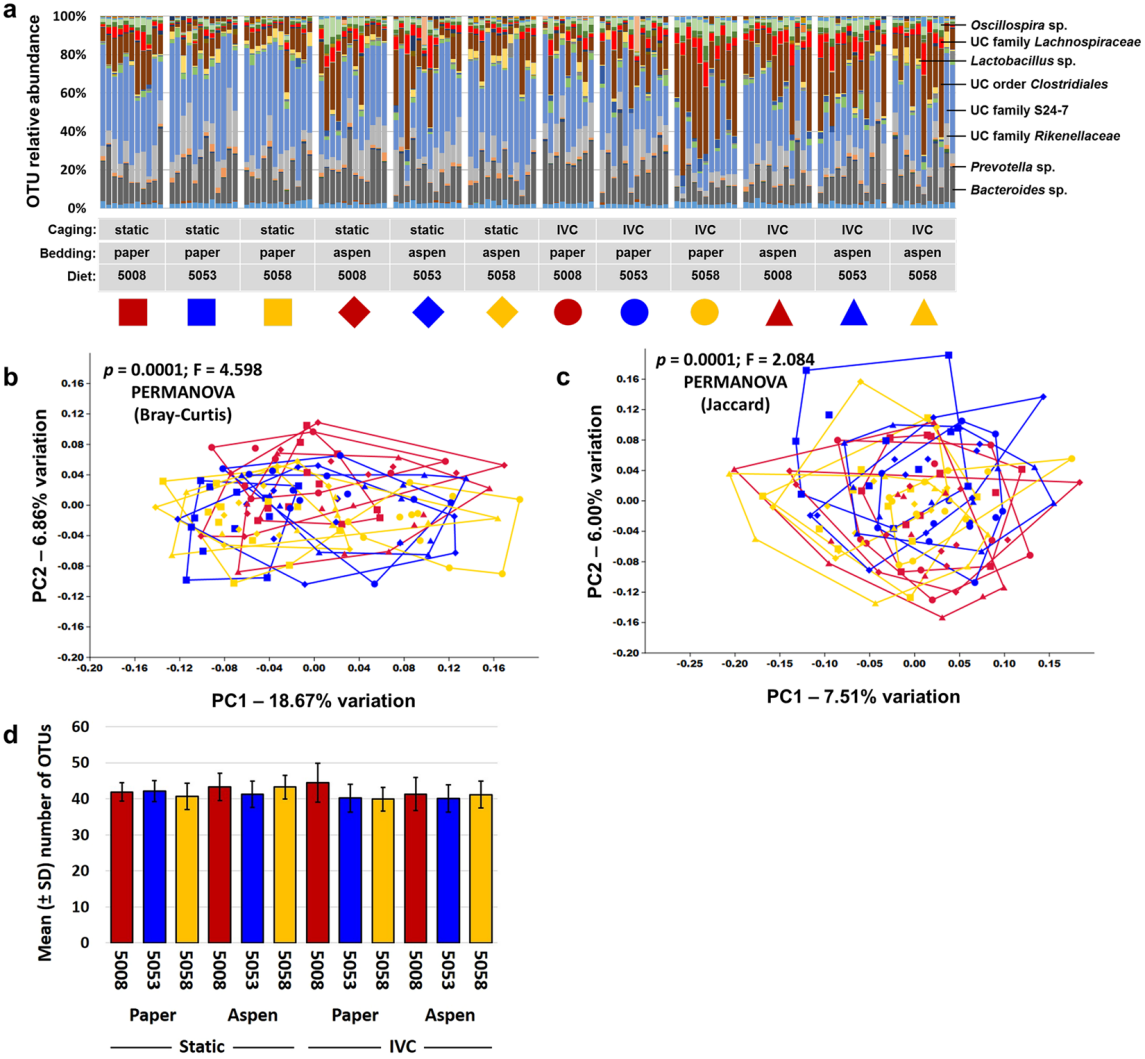
Baseline fecal microbiome is consistent among groups. (**a**) Stacked bar chart showing relative abundance of all operational taxonomic units (OTUs) detected in the feces of 4.5 week-old mice housed in static or ventilated (IVC) microisolator caging, with paper or aspen bedding, and fed one of three standard rodent chows (5008, 5053, or 5058), one week after arrival, identity of prevalent operational taxonomic units (OTUs) at top right; (**b**,**c**) principal coordinate analysis of the samples shown in (**a**) demonstrating the compositional heterogeneity of the baseline fecal microbiota as determined using both Bray-Curtis (**b**) and Jaccard (**c**) distances, legend above. P values indicate results of pairwise comparisons shown in Supplementary Tables S1 and S2 respectively; (**d**) bar chart showing mean ± SD number of OTUs detected at baseline. Combinations of colors and symbols are used to identify factors: red = 5008; blue = 5053; yellow = 5058; squares = static microisolators with paper bedding; diamonds = static microisolators with aspen bedding; circles = IVCs with paper bedding; triangles = IVCs with aspen bedding.

Mice were randomly assigned to each of the various combinations of independent variables (i.e., caging, bedding, and diet) and allowed to acclimate for one week prior to collection of a baseline fecal sample. Twelve weeks later, representing a common study duration in contemporary research, mice were euthanized and luminal contents of the jejunum, ileum, and cecum, and an end-point fecal sample were collected. Following extraction of DNA, PCR was used to generate libraries of amplicons representing the V4 region of the 16S rRNA gene. These libraries were sequenced using the Illumina MiSeq platform, resulting in amplification of 138 of 144 baseline fecal samples (95.8%) to a threshold of 5000 sequences. Based on independent rarefaction of samples from each sample site and time point (i.e., baseline feces, jejunum, ileum, cecum, feces at necropsy), coverage of greater than 5000 sequences resulted in coverage-independent estimates of richness (Supplementary Fig. 1). The mean (±s.d.) coverage for the baseline fecal samples was 42624 (±12009) sequences per sample, resulting in the resolution of, on average, 41.6 (±3.8) operational taxonomic units (OTUs) per sample. In agreement with previous studies, the baseline fecal microbiota was dominated by bacteria in the phyla *Bacteroidetes* and *Firmicutes*, although the phyla *Actinobacteria*, *Cyanobacteria*, *Deferribacteres*, *Proteobacteria*, TM7, and *Tenericutes* were all detected at a low relative abundance in greater than 75% of samples. Bacteria in the phylum *Verrucomicrobia* were detected in 39.86% (55/138) of the baseline fecal samples. Perhaps reflecting the relatively young age of these mice at the time of arrival, the fecal profiles, resolved to the level of OTU, were somewhat inconsistent both between and within treatment groups at one week after arrival (Fig. 1a). While principal coordinate analysis (PCoA) based on either the Bray-Curtis (Fig. 1b) or Jaccard (Fig. 1c) distances and classification based on a hierarchical clustering algorithm (Supplementary Fig. 2) all failed to demonstrate any distinct clustering of treatment groups, a few significant differences were detected between individual groups when tested via one-way PERMANOVA and corrected for multiple testing to account for the high number of pairwise comparisons (Supplementary Tables S1 and S2). PERMANOVA is a non-parametric statistical test for differences between multivariate datasets in the centroid or dispersion of groups, and can be applied to the same dataset using different inter-sample distance metrics. Jaccard distances are based on the agreement between two samples with regard to the presence or absence of OTUs, whereas Bray-Curtis distances also account for the agreement between samples with regard to the relative abundance of shared taxa. No main effects of caging (*p* = 0.169), bedding (*p* = 0.760), or diet (*p* = 0.050) on richness were detected (Fig. 1d). Thus, while we cannot rule out the possibility that one week of housing under the various combinations of fixed variables may have induced the subtle differences detected via PERMANOVA, those differences were relatively minor.

### Endpoint composition of fecal microbiota is relatively unaffected

**Figure 2:**
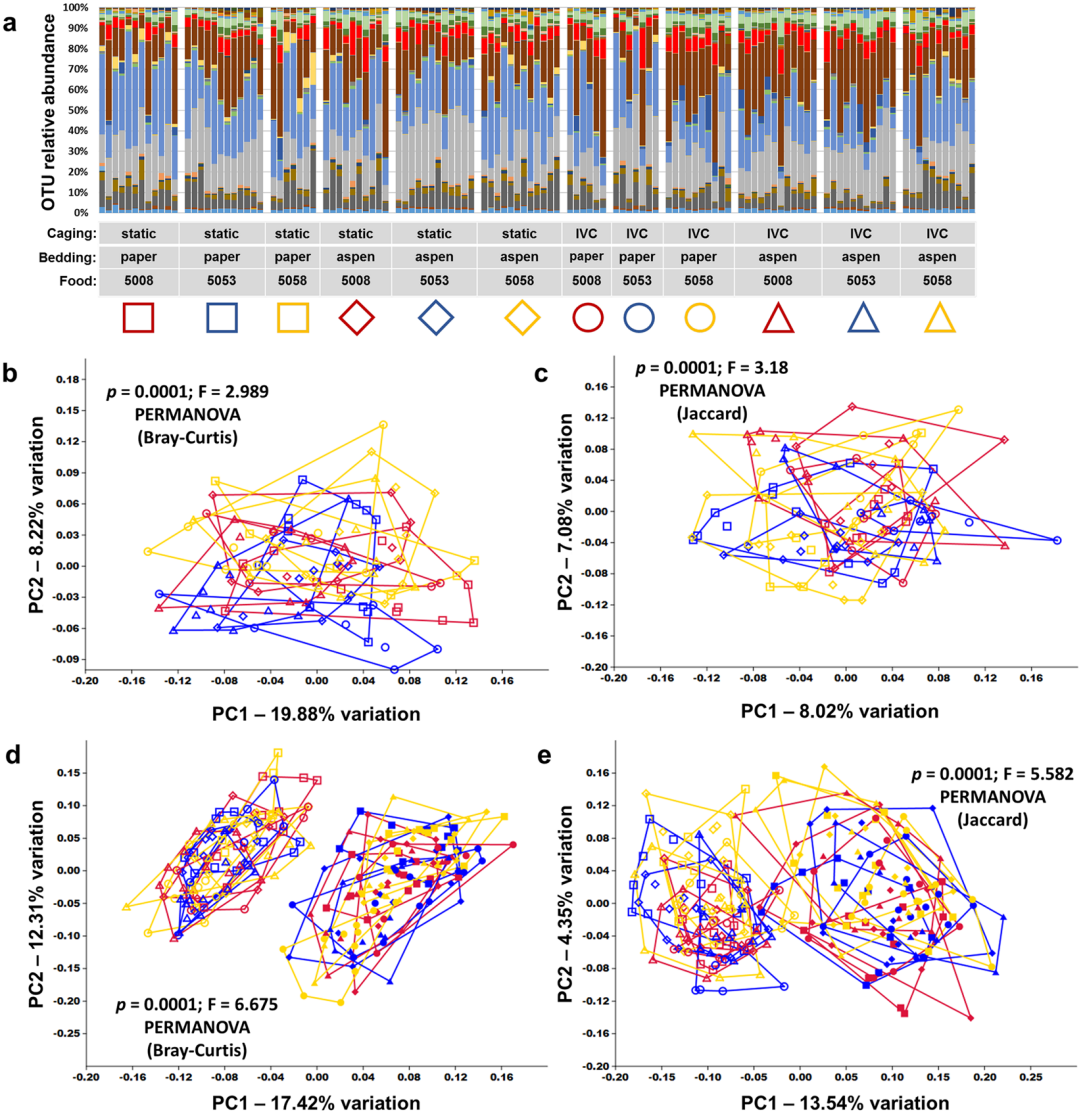
Caging, bedding, and diet have minimal effect on fecal microbiota. (**a**) Stacked bar chart showing relative abundance of all operational taxonomic units (OTUs) detected in the feces of 16.5 week-old mice housed in static or ventilated (IVC) microisolator caging, with paper or aspen bedding, and fed one of three standard rodent chows (5008, 5053, or 5058), thirteen weeks after arrival; (**b**,**c**) principal coordinate analysis (PCoA) of the samples shown in (**a**) demonstrating the compositional heterogeneity of the end-point fecal microbiota as determined using both Bray-Curtis (**b**) and Jaccard (**c**) distances, legend above. P values indicate main effect, results of pairwise comparisons shown in Supplementary Tables S3 and S4 respectively; (**d**,**e**) combined PCoA of the baseline and endpoint fecal samples demonstrating the overall shift in microbial composition of all treatment groups over time based on Bray-Curtis (**d**) and Jaccard (**e**) distances, with no apparent clustering by treatment. Combinations of colors and symbols are used to identify factors: red = 5008; blue = 5053; yellow = 5058; squares = static microisolators with paper bedding; diamonds = static microisolators with aspen bedding; circles = IVCs with paper bedding; triangles = IVCs with aspen bedding.

After 12 additional weeks under the various housing conditions, mice were humanely euthanized and an endpoint fecal sample was collected. Again, subjective assessment of the relative distribution of OTUs as shown via bar charts (Fig. 2a), and overall compositional similarities as visualized via PCoA based on both Bray-Curtis (Fig. 2b) and Jaccard (Fig. 2c) distance matrices, failed to identify consistent commonalities correlating with treatment group, suggesting that caging, bedding, and diet had little effect on the overall composition of fecal bacterial communities. There was however a shift in the composition of the fecal microbiota over the course of the study in all mice (Fig. 2d,e), suggesting that the GM composition had changed over time as the mice aged. Not surprisingly, comparison of initial and final fecal communities detected a significant difference (*p* = 0.0001; F = 6.75 based on Bray-Curtis and *p* = 0.0001; F = 5.582 based on Jaccard, PERMANOVA). Pairwise comparisons within endpoint fecal samples based on the Bray-Curtis distances however, when corrected for multiple testing, detected only two significant differences between groups (out of 66 comparisons) in fecal community composition (Supplementary Table S3). Pairwise comparisons of endpoint fecal communities based on Jaccard distances detected a greater number of significant differences (Supplementary Table S4). Fecal richness and α-diversity were also evaluated via the number of OTUs and Chao1 indices (richness) and Shannon and Simpson indices (α-diversity), and main effects of caging, bedding, and diet were assessed using a 3-way ANOVA implemented in a general linear model (Table 1). Main effects of dietary formulation were detected in Shannon and Simpson indices, but not OTU count or Chao1 index, suggesting that while the various diets tested may affect the distribution of fecal taxa, there is negligible effect on overall richness. There was also a smaller main effect of caging detected on OTU count and subtle interactions between caging and diet on all four metrics, the significance of which are unclear.

**Table 1:** P and F values associated with main effects of, and interactions between, caging, bedding, and diet on the fecal microbial richness and α-diversity following 13 weeks of housing. Mean values and standard deviation within each group are provided in Supplementary Table S5.

		OTU count		Chao1		Shannon		Simpson	
		*p* val	F val	*p* val	F val	*p* val	F val	*p* val	F val
Main effects	Caging	0.004	8.64	0.079	3.15	0.827	0.05	0.207	1.61
	Bedding	0.257	1.30	0.062	3.55	0.067	3.43	0.279	1.18
	Diet	0.994	0.01	0.632	0.46	<0.001	10.84	<0.001	22.78
Interactions	Cage × Bed	0.033	4.65	0.469	0.53	0.163	1.98	0.239	1.40
	Cage × Diet	0.004	5.82	0.003	6.23	0.002	6.70	0.016	4.29
	Bed × Diet	0.626	0.47	0.671	0.40	0.987	0.01	0.270	1.33
	C × B × D	0.927	0.08	0.532	0.63	0.182	1.73	0.317	1.16

### Endpoint composition of jejunal, ileal, and cecal microbiota reveals husbandry-associated differences

**Figure 3:**
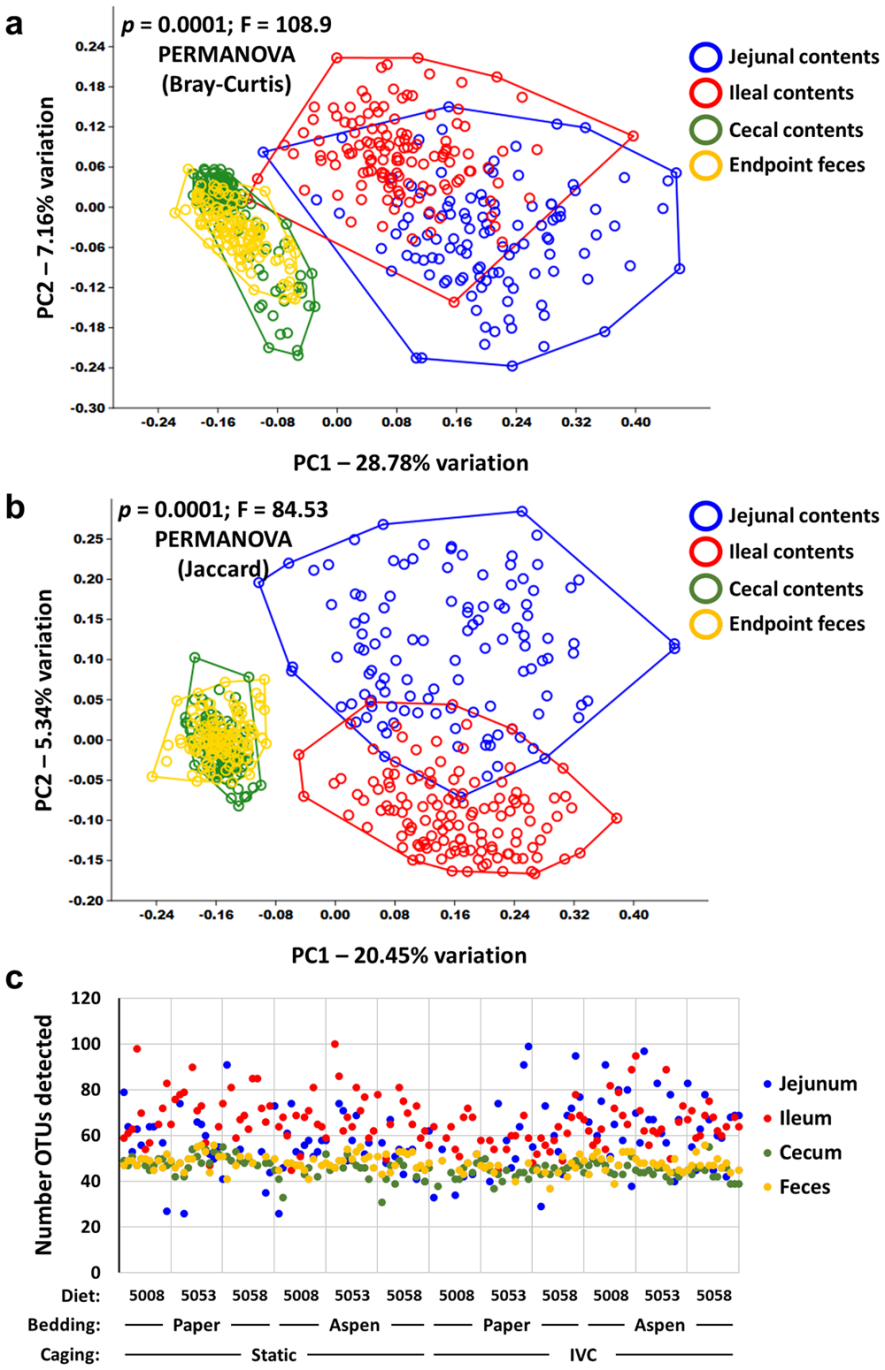
Contents of small and large intestines differ in composition and richness. (**a**,**b**) Principal coordinate analysis of the jejunal contents, ileal contents, cecal contents, and endpoint fecal samples of 16.5 week-old mice housed under various housing conditions for thirteen weeks, based on the Bray-Curtis (**a**) and Jaccard (**b**) distances; (**c**) dot plot showing the number of operational taxonomic units (OTUs) detected in samples from each region, for each individual mouse.

At necropsy, the luminal contents of other regions of the GIT were collected and processed for 16S rRNA amplicon sequencing to determine whether the endpoint fecal microbiota was representative of the entire gut, and whether any of the treatments influenced the composition of microbiota present in other anatomic regions. Perhaps not surprisingly, the microbial composition of the various regions were markedly different from each other with the most prominent distinction occurring between the small and large intestines. Regarding similarities in the overall composition of the microbiota, PCoA resulted in a clear separation of the upper (i.e., jejunum and ileum) and lower (i.e., cecum and feces) GIT along principal coordinate 1 (PC1) which described 28.78% and 20.45% of the variation in the Bray-Curtis (Fig. 3a) and Jaccard (Fig. 3b) distance matrices. Regardless of the distance metric used, the jejunal contents and ileal contents separated, albeit incompletely, from each other along PC2 and both groups of samples demonstrated a greater overall spread indicating a wider range of possible compositions within those regions of the GIT. In contrast, the cecal and fecal microbial communities did not separate along PC1 or PC2 (Fig. 3a,b), suggesting that these regions harbor similar bacterial communities. That said, significant differences were found between all pairwise comparisons (*p* < 0.0001), with F values ranging from 36.71 (between cecal contents and feces) to 207.8 (between cecal and jejunal contents). Comparison of the richness of samples from each region revealed a similar division between the upper and lower GIT with a mean (±SEM) of 58.9 (±1.4), 66.0 (±1.0), 45.7 (±0.4), and 48.1 (±0.3) OTUs detected in the jejunal, ileal, cecal, and endpoint fecal samples respectively (Fig. 3c). Kruskal-Wallis one way ANOVA on ranks with post hoc comparison via Dunn’s method detected significant differences between all pairwise comparisons.

**Figure 4:**
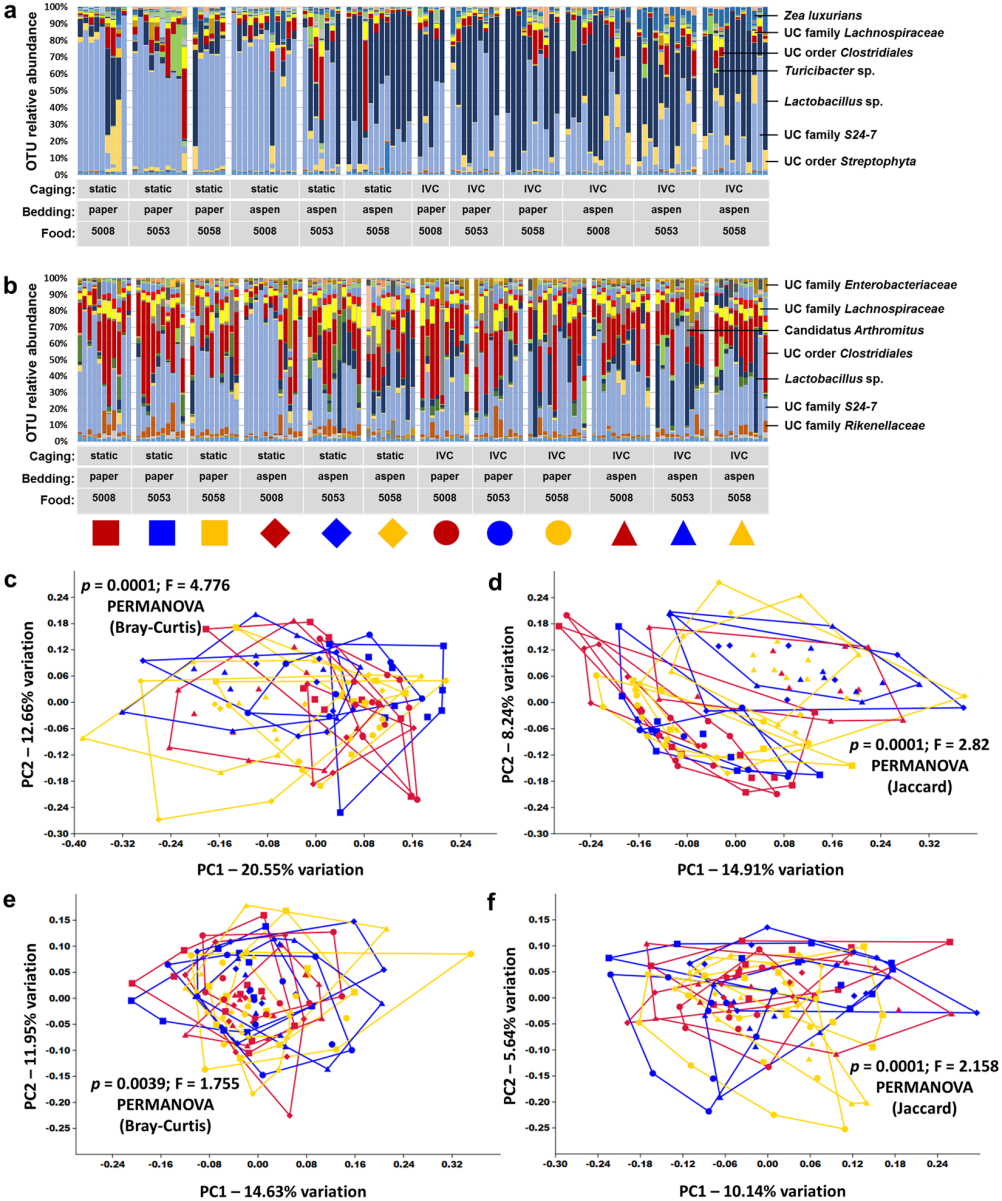
Caging, bedding, and diet have minimal effect on small intestinal microbiota. (**a**,**b**) Stacked bar charts showing relative abundance of all operational taxonomic units (OTUs) detected in the jejunal (**a**) and ileal (**b**) contents of 16.5 week-old mice housed in static or ventilated (IVC) microisolator caging, with paper or aspen bedding, and fed one of three standard rodent chows (5008, 5053, or 5058), thirteen weeks after arrival, identity of prevalent operational taxonomic units (OTUs) at top right; (**c**,**d**) principal coordinate analysis (PCoA) of the samples shown in (**a**) based on Bray-Curtis (**c**) and Jaccard (**d**) distances, legend above; (**e**.**f**) PCoA of the samples shown in (**b**), based on Bray-Curtis (**e**) and Jaccard (**f**) distances. All *p* values indicate main effect, results of corresponding pairwise comparisons shown in Supplementary Tables S6 through S9. Combinations of colors and symbols are used to identify factors: red = 5008; blue = 5053; yellow = 5058; squares = static microisolators with paper bedding; diamonds = static microisolators with aspen bedding; circles = IVCs with paper bedding; triangles = IVCs with aspen bedding.

When the microbiota detected in each region of the gut was considered independently, several interesting trends were noted. Considering first the jejunal contents, the majority of samples were dominated by microbes in the poorly characterized family S24-7, and *Lactobacillus* sp. (Fig. 4a). Other OTUs detected in high relative abundance in multiple samples included sequences annotated to the order *Streptophyta* and *Zea luxurians* (presumably due to homology to mitochondria-specific sequences present in dietary plant matter as reported elsewhere^19^), unidentified microbes in the order *Clostridiales*, family *Lachnospiraceae*, and *Turicibacter* sp. While there were several similarities between ileal and jejunal contents, the brief transit time between regions nonetheless resulted in distinct microbial populations. OTUs that were generally reduced in relative abundance in ileal contents, relative to the jejunum, include *Lactobacillus* sp. and microbes in the order *Streptophyta* (Fig. 4b). These decreases were compensated by overall increases in the relative abundance of unclassified microbes in the order *Clostridiales*, and families *Enterobacteriaceae*, *Lachnospiraceae*, and *Rikenellaceae*. Additionally, segmented filamentous bacteria (SFB) in the candidate genus *Arthromitus* (i.e., Candidatus *Arthromitus*) were intermittently detected at a high relative abundance of several ileal samples. These microbes are probably more properly classified as Candidatus *Savagella*, the genera of SFB found in mammalian hosts^20,21^. PCoA of jejunal samples demonstrated variable clustering depending on the distance matrix used (Fig. 4c,d). PERMANOVA based on the Bray-Curtis distance matrix detected a small number of significant differences between the jejunal microbiota present in the 12 treatment groups (Supplementary Table S6); the parallel analysis based on the Jaccard distances detected a greater number of differences (Supplementary Table S7) suggesting that the differences are driven by discrepancies in community membership rather than differences in relative abundance of shared taxa. No significant differences were found when comparing ileal communities (Supplementary Tables S8 and S9). Collectively, the above data suggest that the husbandry-associated variables under study influence the composition of the microbiota present in the upper GIT.

**Figure 5:**
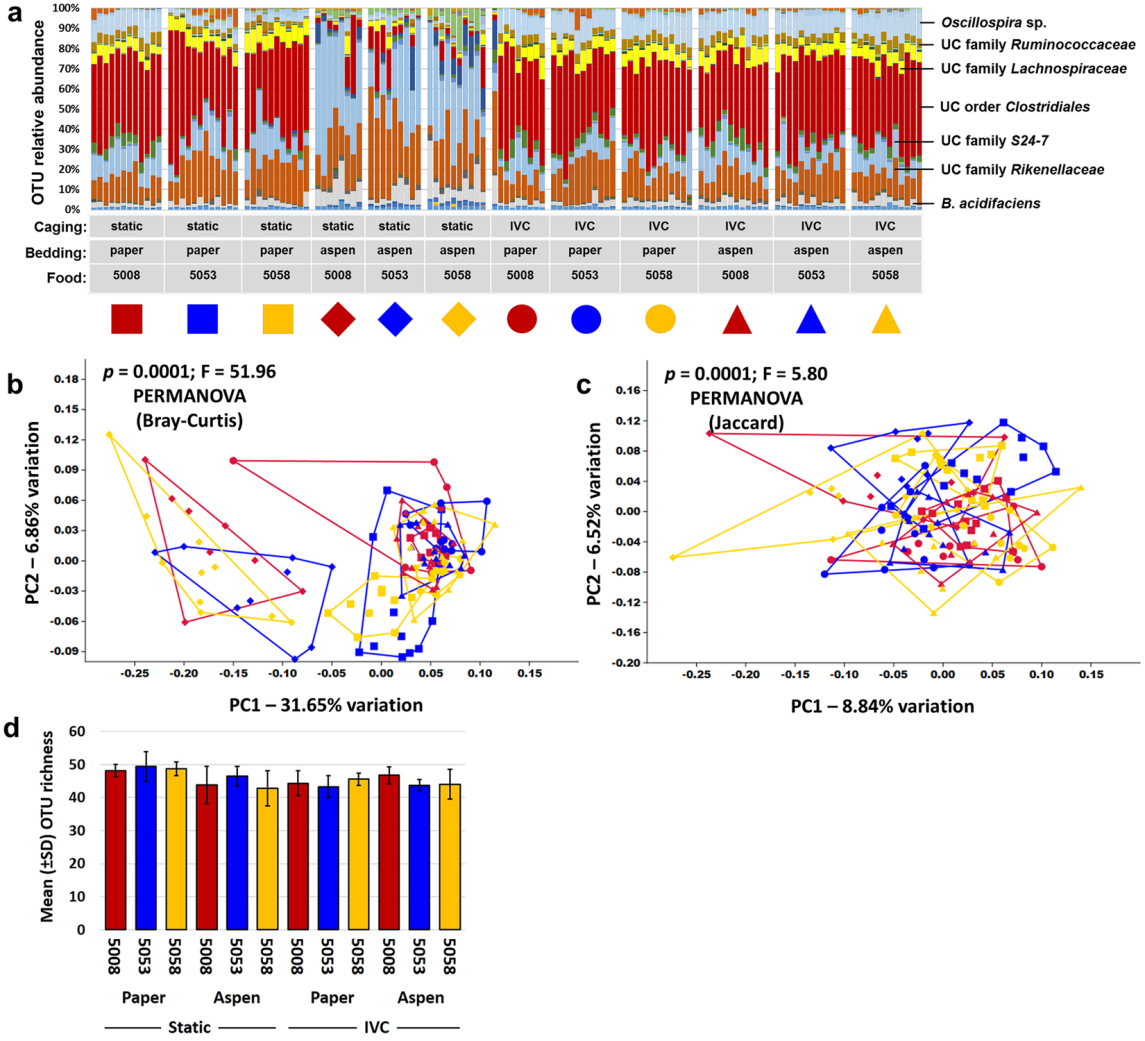
Interactions between caging and bedding have strong effect on cecal microbiota. (**a**) Stacked bar chart showing relative abundance of all operational taxonomic units (OTUs) detected in the cecal contents of 16.5 week-old mice housed in static or ventilated (IVC) microisolator caging, with paper or aspen bedding, and fed one of three standard rodent chows (5008, 5053, or 5058), thirteen weeks after arrival; (**b**,**c**) principal coordinate analysis (PCoA) of the samples shown in (**a)** demonstrating the difference in cecal microbiota of mice housed under the various conditions tested as determined using both Bray-Curtis (**b**) and Jaccard (**c**) distances, legend above. All *p* values indicate main effect, results of pairwise comparisons shown in Supplementary Tables S10 and S11 respectively; (**d**) bar chart showing mean ± SD number of OTUs detected in cecal contents. Combinations of colors and symbols are used to identify factors: red = 5008; blue = 5053; yellow = 5058; squares = static microisolators with paper bedding; diamonds = static microisolators with aspen bedding; circles = IVCs with paper bedding; triangles = IVCs with aspen bedding.

Unexpectedly, similar analysis of the cecal contents revealed several remarkable characteristics. First and foremost, there was a clear and consistent difference between mice housed in static caging with aspen bedding relative to all other groups in the composition of the cecal microbiota (Fig. 5a). The majority of mice housed in the combination of static caging and aspen bedding retained a high relative abundance of unclassified microbes in the family S24-7, and most still harbored the sizeable proportions of *Lactobacillus* sp. seen in the ileum. These groups also demonstrated substantially greater relative abundance of *Bacteroides acidifaciens*. Alternatively, all other groups of mice showed consistent and notable increases in the relative abundance of unclassified microbes in the order *Clostridiales*, families *Lachnospiraceae* and *Ruminococcaceae* (Clostridium clusters XIVa and IV respectively), and *Oscillospira* sp. while these taxa were detected at much lower relative abundance in the aforementioned mice (Supplementary Figure S3). Also of note is the finding that samples from mice housed in ventilated racks (all groups) clustered more closely together than those housed in static microisolators suggesting that ventilated housing may be more optimal for studies that require consistency in the GM. As might be surmised from the bar charts, PCoA of the cecal contents revealed negligible effect of the three dietary formulations tested but a clear separation of the three groups of mice housed in static caging with aspen bedding from the other nine groups along PC1, which explained 31.65% and 8.84% of the variation depending on the distance metric used (Figs. 5b,c). Interestingly, the other three groups of mice housed in static caging (with paper bedding) showed a partial separation from the six groups of mice housed in IVC systems, and those mice housed in IVC systems did not cluster independently at all regardless of bedding. Data visualized across the first three principal coordinates revealed an identical picture (Supplementary Video S1). As anticipated, PERMANOVA of Bray-Curtis and Jaccard distances detected many significant differences (Supplementary Tables S10 and S11). While richness did not vary substantially between groups (Fig. 5d), α-diversity indices showed a similar pattern to PCA wherein mice housed in static microisolator cages with aspen bedding demonstrated lower diversity (Supplementary Figure S4). Significant main effects of each variable on cecal richness and diversity were detected, as were strong interactions between caging and bedding (Table 2).

**Table 2:** P and F values associated with main effects of, and interactions between, caging, bedding, and diet on the cecal microbial richness and α-diversity following 13 weeks of housing. Mean values and standard deviation within each group are provided in Supplementary Table S12.

		OTU count		Chao1		Shannon		Simpson	
		*p* val	F val	*p* val	F val	*p* val	F val	*p* val	F val
Main effects	Caging	0.002	9.96	0.009	7.10	<0.001	69.54	<0.001	32.40
	Bedding	0.002	10.19	<0.001	71.94	<0.001	24.06	0.070	3.35
	Diet	0.766	0.27	0.05	3.08	0.007	5.18	0.001	7.11
Interactions	Cage × Bed	<0.001	15.19	<0.001	89.47	<0.001	53.59	0.001	11.05
	Cage × Diet	0.021	4.01	0.003	6.25	0.715	0.34	0.012	4.56
	Bed × Diet	0.126	2.11	0.524	0.65	0.692	0.37	0.724	0.32
	C × B × D	0.554	0.59	0.464	0.77	0.041	3.28	0.075	2.65

**Figure 6:**
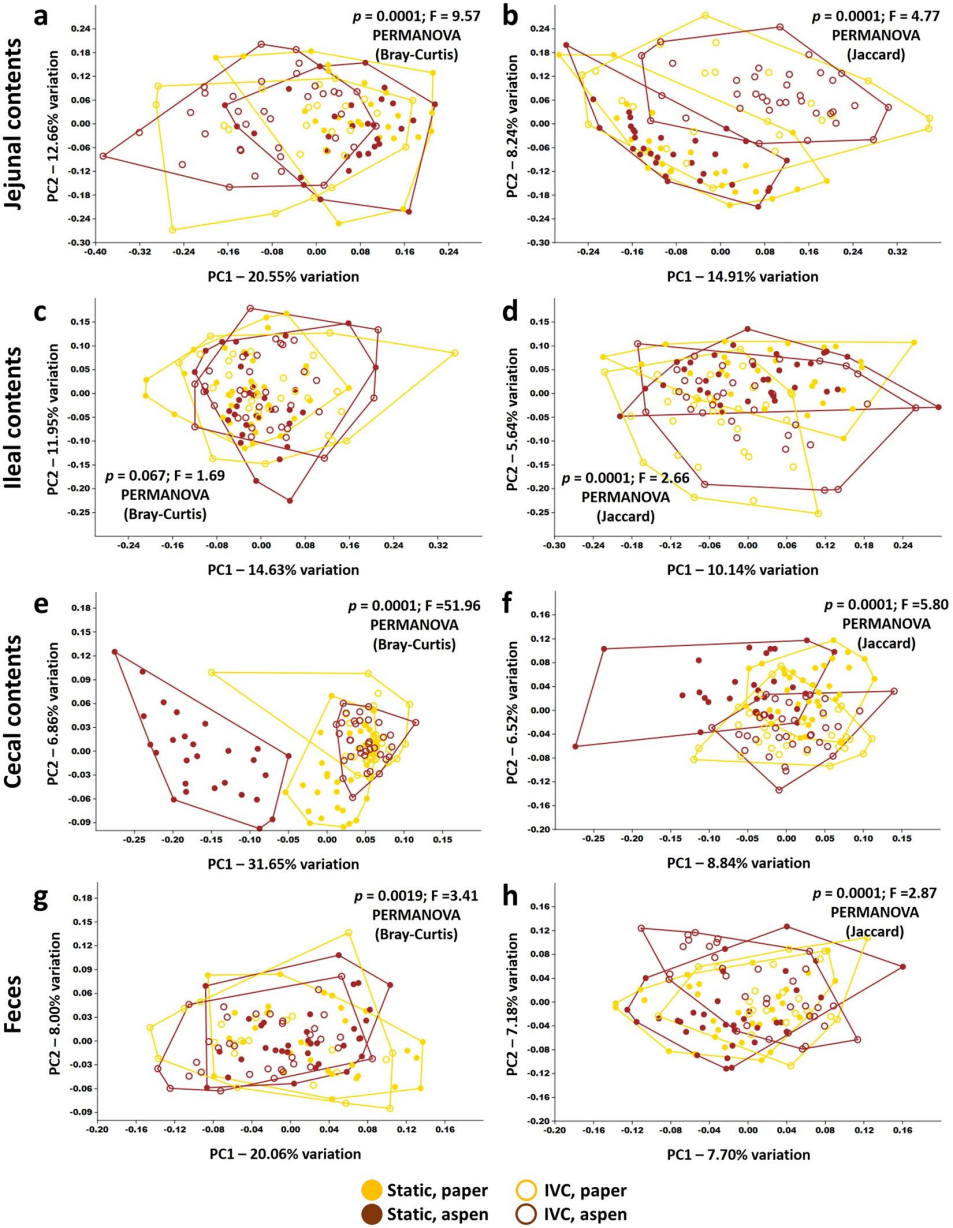
Combined effects of caging and bedding on microbiota muted in fecal samples. (**a**,**b**) Principal coordinate analysis (PCoA) of jejunal microbiota of mice grouped according to their combination of static or IVC housing and paper or aspen bedding, as determined using both Bray-Curtis (**a**) and Jaccard (**b**) distances, legend at bottom; (**c**–**h**) PCoA of ileal (**c**,**d**), cecal (**e**,**f**), and fecal (**g**,**h**) microbiota from the same mice, as determined using Bray-Curtis (**c**,**e**,**g**) and Jaccard (**d**,**f**,**h**) distances.

Visualizing each anatomic site separately based on the combination of housing and bedding, the jejunal and cecal contents appear to be the most affected while fecal communities are least divergent between mice housed under different condition (Fig. 6). Collectively, these data indicate that the husbandry used in laboratory animal care can have a strong influence on the composition and intra-group variability of the cecal microbiota, and that those influences can be due to interactions between multiple variables, such as cage type and bedding as seen here. Viewed in the context of the jejunal, ileal, and fecal microbial profiles, these data also suggest that the cecum represents a unique niche in gastrointestinal microbial ecology. While maintaining a highly uniform profile in the majority of treatment groups, the cecum was the primary anatomic site wherein environmental influences were reflected in the composition of the microbiota.

## Discussion

The data presented above carry several implications for animal modeling, particularly in the context of research investigating the gut microbiota. At the most basic level, our findings provide proof-of-concept that seemingly benign factors associated with rodent husbandry can influence the composition and heterogeneity of the resident microbiota in certain gut regions. Thoene-Reineke *et al*. previously demonstrated that housing several strains of immunodeficient mice in either ventilated racks or open top caging resulted in subtle changes to their fecal microbiota^22^. Curiously, such changes were not seen in wild type mice. While our studies used a slightly different design (commonly used immunocompetent, outbred stock of mice and ventilated racks vs. static microisolators), we also found no differences in fecal microbiota of wild type mice housed in different settings. However, we found unexpected stark differences in the cecal microbiota of mice subjected to different housing conditions. Moreover, the observed differences in the cecal microbiota were dependent on an interaction of two variables, cage ventilation and bedding, and no differences were detected in the cecal microbiota of mice exposed to only one of the two factors in question. One possible explanation for this bedding/housing interaction effect is the presence of an unknown aromatic or volatile compound in aspen bedding that is removed from ventilated microisolator cages but remains in static cages at a level capable of influencing the cecal microbiota. Any such compound could be exogenous in nature or may be of host-origin, such as urinary ammonia absorbed differentially by the two types of bedding. In addition to removal of volatile compounds, the increased noise or airflow of IVCs have also been shown to be a potential stress factor for mice^23^ which may influence GM composition. Collectively, our findings in conjunction with those of previous studies^22^ highlight the complexity by which factors (e.g. mouse strain, housing/ventilation type, bedding and sample location) may influence intestinal microbiota.

Comparisons of coliform counts and lipopolysaccharide (LPS) levels in various types of rodent bedding, performed using standard microbiological techniques and fluorometric assays respectively, suggest that there are biologically relevant differences, with hardwood and corncob beddings containing significantly greater levels of LPS relative to paper^4,24^, and corncob, hardwood, and paper bedding containing high, moderate, and low coliform counts respectively^4^. Those findings may partially explain differences between mice housed on hardwood and cotton bedding in total IgA production, although the specificity of the enhanced mucosal immune responses by mice housed on hardwood bedding is not clear^25^. In combination with other data suggesting the potential of microbial contamination of corncob bedding, these findings suggest that corncob and hardwood beddings may be more likely sources of environmental microbial contamination than paper or cotton products. Similarly, numerous studies have investigated the impact of housing density^8,26–29^, cage change frequency^6,29,30^, caging system and ventilation^31,32^, and other husbandry-related variables from the standpoint of animal well-being but very few have explored the influence of these variables on the GM composition.

The present data also suggest that cecal contents may be a better indicator of environmental influences on GM, and the use of fecal samples may lead to “false negatives” when screening for effects on the GM. The reason for this is unknown but the microbial composition within the lumen of the GI tract apparently normalizes during colonic transit. While the use of feces as the representative sample provides several obvious benefits such as non-invasive acquisition (thus allowing longitudinal studies with repeated measures), collection and analysis of cecal contents should also be considered in terminal studies, particularly those investigating the effects of environmental influences on the GM. Alternatively, longitudinal studies incorporating analysis of cecal communities could be performed using separate cohorts of mice taken down at intervals.

Considering recent calls by the NIH for measures to enhance the reproducibility of preclinical research^33,34^, particularly studies employing animal models, these and other data^35^ demonstrate the need to consider the gut microbiota, and by extension, the husbandry of research animals. Researchers at different institutions performing the same experiments, with the same strain of mouse purchased from the same vendor, could generate different data and reach ostensibly different conclusions. Both could be completely valid datasets, the difference merely reflecting a microbial influence on experimental outcomes. In addition to the differences in the composition of the cecal microbiota between mice housed in static caging with aspen bedding and the other groups, there was also an apparent difference in the uniformity of the cecal microbiota. As sample size calculations are partially based on the anticipated variance present in the outcome of interest, it is reasonable to believe that decreased variance in the composition of the GM through certain husbandry practices (e.g., mice housed in ventilated housing in the current study) could decrease the sample size needed to achieve adequate study power.

In conclusion, this study demonstrates that differences in factors such as caging and bedding can modulate the gut microbiota and that cecal samples might be optimal when screening for environmental effects on the gut microbiota. Ultimately, whether or not such changes in the composition of the microbiota have downstream effects on model phenotypes and associated study reproducibility will have to be determined on an individual basis but the possibility must be considered by researchers.

## Methods

### Mice

Four week-old female outbred Crl:CD1 (ICR) mice (*N* = 144) were purchased from Charles River Laboratories (Wilmington, MA) in a single order, and housed in the same room and maintained under barrier conditions in microisolator cages on free-standing shelves or on individually ventilated cage-racks (Thoren, Hazleton, PA), filled with either compressed paper (Paperchip® Brand Laboratory Animal Bedding, Shepherd Specialty Papers, Watertown, TN) or aspen chip bedding (Aspen Chip® Aspen Hardwood Laboratory Bedding, Northeastern Products Corp., Warrensburg, NY), with *ad libitum* access to autoclaved rodent chow (LabDiet 5008, 5053, or 5058, Purina, St. Louis, MO) and acidified, autoclaved water, under a 14:10 light/dark cycle. Each treatment group representing a different combination of caging, bedding, and diet comprised three cages, and all cages contained a nestlet for enrichment and four mice per cage. Water was acidified using an automated bottle filler (model 9WEF, Tecniplast, Buguggiate, Italy) designed to titrate municipal water with sulfuric acid to a target pH of 2.5 (range 2.3 to 2.7). Mice were determined to be free of all overt and opportunist bacterial pathogens including *Citrobacter rodentium*, *Helicobacter* spp., *Mycoplasma* spp., *Pasteurella pneumotropica*, *Pseudomonas aeruginosa*, *Salmonella* spp., *Staphylococcus aureus*; *Encephalitozoon cuniculi*; adventitious viruses including MHV, MVM, MS1 (generic parvo), MPV, MNV, TMEV, EDIM, Sendai, PVM, REO3, LCMV, Ectromelia, MAV1, MAV2, and polyoma viruses; intestinal protozoa including *Spironucleus muris*, *Giardia muris*, *Entamoeba muris*, *Tritrichomonas muris*, and other large intestinal flagellates and amoeba; intestinal parasites including pinworms and tapeworms; and external parasites including all species of lice and mites, via quarterly sentinel testing performed by IDEXX BioResearch (Columbia, MO). Mice were allowed to acclimate for a period of one week prior to the initial sample collection. At the end of study, mice were humanely euthanized via inhaled carbon dioxide, in accordance with the AVMA Guidelines for the Euthanasia of Animals: 2013 Edition, followed by cervical dislocation as a secondary means. All procedures were performed in accordance with the Guide for the Care and Use of Laboratory Animals and under approval of the University of Missouri Institutional Animal Care and Use Committee.

### Sample collection

Two freshly evacuated fecal pellets were obtained from each mouse at 1 week post-arrival. These samples were collected by opening each microisolator cage in a class II biological safety cabinet, transferring each mouse to a separate clean microisolator cage containing no bedding, and allowing the mouse to defecate normally. Fecal pellets were then collected with a sterile wooden toothpick. Twelve (12) weeks later, mice were humanely euthanized and jejunal, ileal, cecal, and fecal samples were collected using aseptic technique. Briefly, each region of the gut was exteriorized to allow collection of samples from roughly the same site of each animal. In addition to collecting luminal contents, the mucosa was gently scraped to ensure that mucosa-associated microbes were included in samples. Jejunal samples were collected from the approximate middle of the jejunum; ileal samples were collected from approximately 3 cm proximal to the ileocecocolic junction; cecal samples comprised the entire cecal contents; and fecal samples represented the most distal fecal bolus present in the GIT, excluding boli within the rectum proper. Instruments used for collection were flamed and allowed to cool between all samples. Following collection, all samples were immediately placed in 2 mL round-bottom tubes containing 800 µL lysis buffer^36^ and a 0.5 cm diameter stainless steel bead. All terminal samples were collected between 7 and 11 a.m. on two consecutive days.

### DNA extraction

Following mechanical disruption using a TissueLyser II (Qiagen, Venlo, Netherlands), tubes were incubated at 70 °C for 20 minutes with periodic vortexing. Samples were then centrifuged at 5000 × g for five minutes at room temperature, and the supernatant transferred to a clean 1.5 mL Eppendorf tube. Two hundred µL of 10 mM ammonium acetate was added to lysates, mixed thoroughly, incubated on ice for five minutes, and then centrifuged as above. The supernatant was then aspirated, mixed thoroughly with one volume of chilled isopropanol, and incubated on ice for 30 minutes. Samples were then centrifuged at 16000 × g for 15 minutes at 4 °C. The supernatant was aspirated and discarded, and the DNA pellet washed several times with 70% ethanol and resuspended in 150 µL of Tris-EDTA. Fifteen µL of proteinase-K and 200 µL of Buffer AL (DNeasy Blood and Tissue kit, Qiagen) were added and samples were incubated at 70 °C for 10 minutes. Two hundred µL of 100% ethanol was added and the contents of each tube were transferred to a spin column from the DNeasy kit. DNA was then purified according to the manufacturer’s instructions and eluted in 200 µL of EB buffer (Qiagen). Yield was determined via fluorometry (Qubit, Life Technologies, Carlsbad, CA) using the quant-iT BR dsDNA reagent kit (Invitrogen, Carlsbad, CA).

### 16S rRNA library preparation and sequencing

Extracted DNA was processed at the University of Missouri DNA Core Facility. Bacterial 16S rDNA amplicons were constructed via amplification of the V4 hypervariable region of the 16S rDNA gene with universal primers (U515F/806 R), flanked by Illumina standard adapter sequences^37,38^. Oligonucleotide sequences are available at proBase^39^. A single forward primer and reverse primers with a unique 12-base index were used in all reactions. PCR reactions (50 µL) contained 100 ng of genomic DNA, forward and reverse primers (0.2 µM each), dNTPs (200 µM each), and Phusion High-Fidelity DNA Polymerase (1U). PCR amplification was performed as follows: 98 °C^(3:00)^ + [98 °C^(0:15)^ + 50 °C^(0:30)^ + 72 °C^(0:30)^] × 25 cycles + 72 °C^(7:00)^. Amplified product (5 µL) from each reaction was combined and thoroughly mixed; pooled amplicons were purified by addition of Axygen AxyPrep MagPCR Clean-up beads to an equal volume of 50 µL of amplicons and incubated at room temperature for 15 minutes. Products were washed multiple times with 80% ethanol and the dried pellet resuspended in Qiagen EB Buffer (32.5 µL), incubated at room temperature for 2 minutes, and then placed on the magnetic stand for 5 minutes. The final amplicon pool was evaluated using the Advanced Analytical Fragment Analyzer automated electrophoresis system, quantified with the Qubit fluorometer using the quant-iT HS dsDNA reagent kit, and diluted according to Illumina’s standard protocol for sequencing on the MiSeq.

### Informatics

Assembly, binning, and annotation of DNA sequences was performed at the MU Informatics Research Core Facility. Briefly, contiguous DNA sequences were assembled using FLASH software^40^, and culled if found to be short after trimming for a base quality less than 31. Qiime v1.8^41^ software was used to perform *de novo* and reference-based chimera detection and removal, and remaining contiguous sequences were assigned to operational taxonomic units (OTUs) via *de novo* OTU clustering and a criterion of 97% nucleotide identity. Taxonomy was assigned to selected OTUs using BLAST^42^ against the Greengenes database^43^ of 16S rRNA sequences and taxonomy. Principal coordinate analyses, performed using ¼ root-transformed OTU relative abundance data, and alpha-diversity indices were determined using the Past 3.15 software package^44^. Heatmaps of log-transformed relative abundance data arranged via hierarchical clustering were generated using Metaboanalyst 3.0^45,46^. The supplementary video was generated using unweighted UniFrac distances and the Qiime script beta_diversity_through_plots.py. Frames of data for animation were created using the R package rgl (r-forge.r-project.org/projects/rgl. ImageMagick (www.imagemagick.org) was used to create the animation.

### Statistics

Differences between groups in richness and alpha-diversity metrics were tested using a three-way analysis of variance (ANOVA) performed via a general linear model in SigmaPlot 13.0. Differences between groups in the relative abundance of 25 independently filtered operational taxonomic units with the highest loading scores were tested using a similar method although data were first quarter-root transformed to normalize for high sparsity. Differences between groups in beta-diversity were determined via one-way permutational multivariate analysis of variance (PERMANOVA^17^) of Bray-Curtis and Jaccard distances using Past 3.15^44^. Jaccard distances are an unweighted metric, based on the shared presence or absence of taxa between samples; Bray-Curtis distances (actually a dissimilarity as it does not satisfy “triangle inequality” but referred to hereafter as a distance metric for the sake of brevity) are weighted and thus factor in similarities in relative abundance of shared taxa. When making pairwise comparisons among all twelve groups, Bonferroni’s method was used to correct for multiple testing.

### Data availability

All reported data have been deposited in the National Center for Biotechnology Information (NCBI) Sequence Read Archive (SRA) under BioProject accession number PRJNA382943.

## Electronic supplementary material

Supplementary Figures and Tables

Supplementary Video S1.
