# Supplementary material for: The influence of caging, bedding, and diet on the composition of the microbiota in different regions of the mouse gut
Source: Sci Rep. 2018 Mar 6;8:4065. doi: 10.1038/s41598-018-21986-7 (PMC5840362; doi:10.1038/s41598-018-21986-7)
Supplement: Supplementary file 1 — Supplementary Figures and Tables [file 41598_2018_21986_MOESM1_ESM.pdf]

Supplementary materials

The influence of caging, bedding, and diet on the composition of the microbiota in different regions of the mouse gut

Aaron C. Ericsson[1,2,3], Jonalyn Gagliardi[3], Delia Bouhan[1], William G. Spollen[4], Scott A. Givan[4], and Craig L. Franklin[1,2,3]*

[1]University of Missouri Mutant Mouse Resource and Research Center

[2]University of Missouri Metagenomics Center

[3]University of Missouri, College of Veterinary Medicine, Department of Veterinary Pathobiology

[4]University of Missouri, Informatics Research Core Facility

*Corresponding author

4011 Discovery Drive, N128

Columbia, MO, 65201

573-882-6623

franklinc@missouri.edu

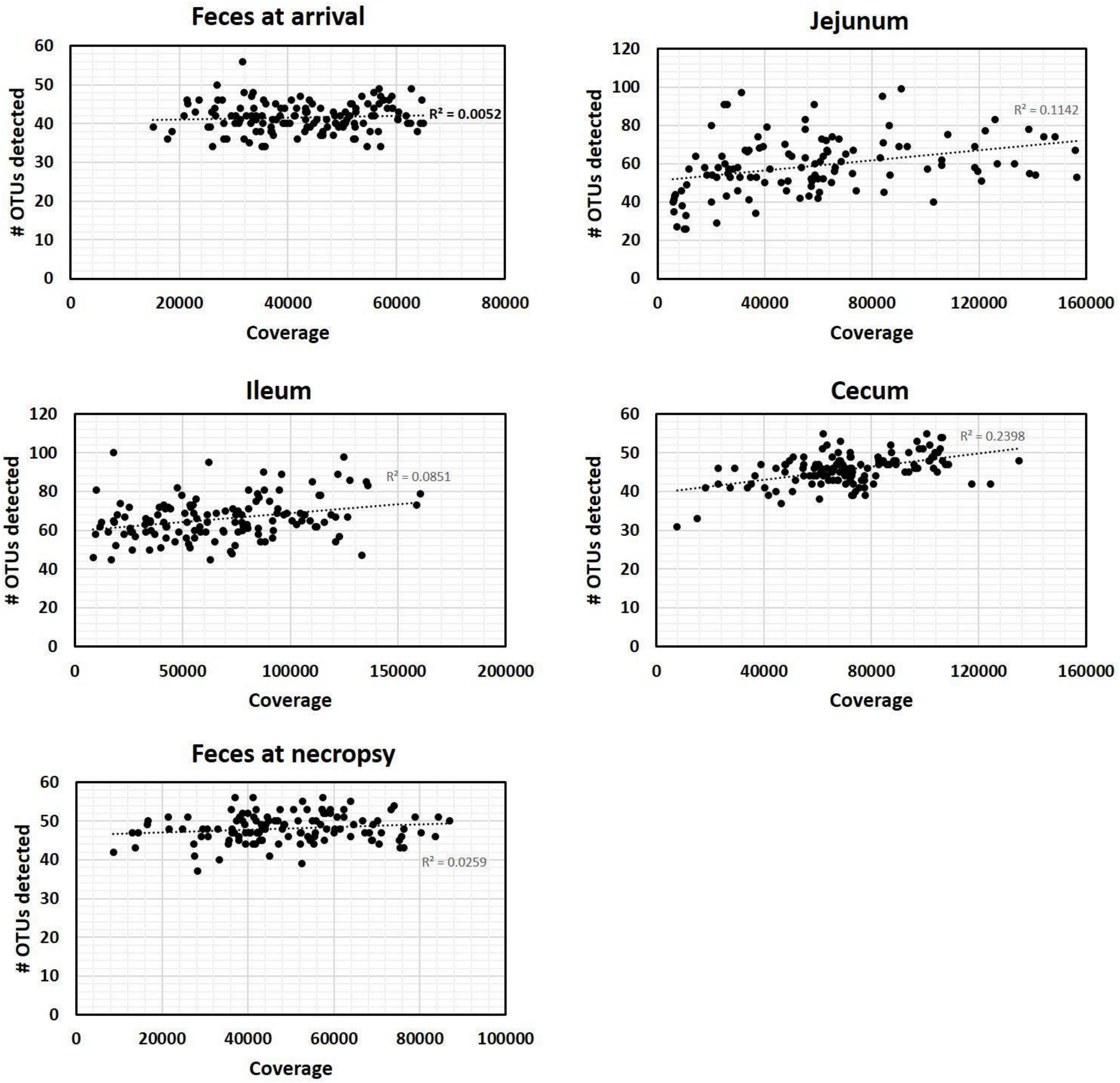

Supplementary Figure S1. Rarefaction of samples collected from each site, showing consistency of detected microbial richness for samples with coverage greater than 5000 sequences per sample.

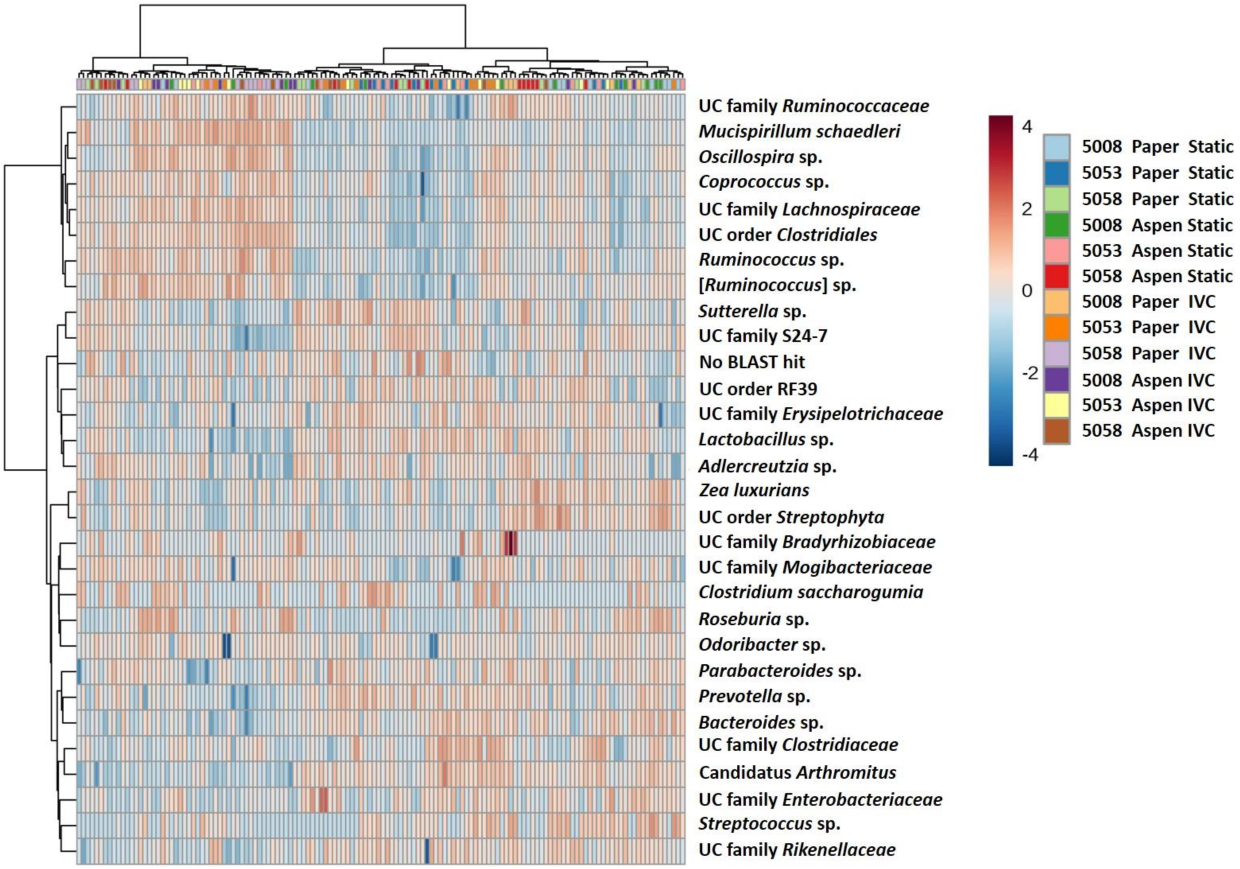

Supplementary Figure S2. Heatmap of log-fold relative abundance in baseline feces of top 25 differentially abundant operational taxonomic units (OTUs) as determined via analysis of variance, arranged via agglomerative hierarchical clustering of Euclidean distances between samples (x-axis) and OTUs (y-axis).

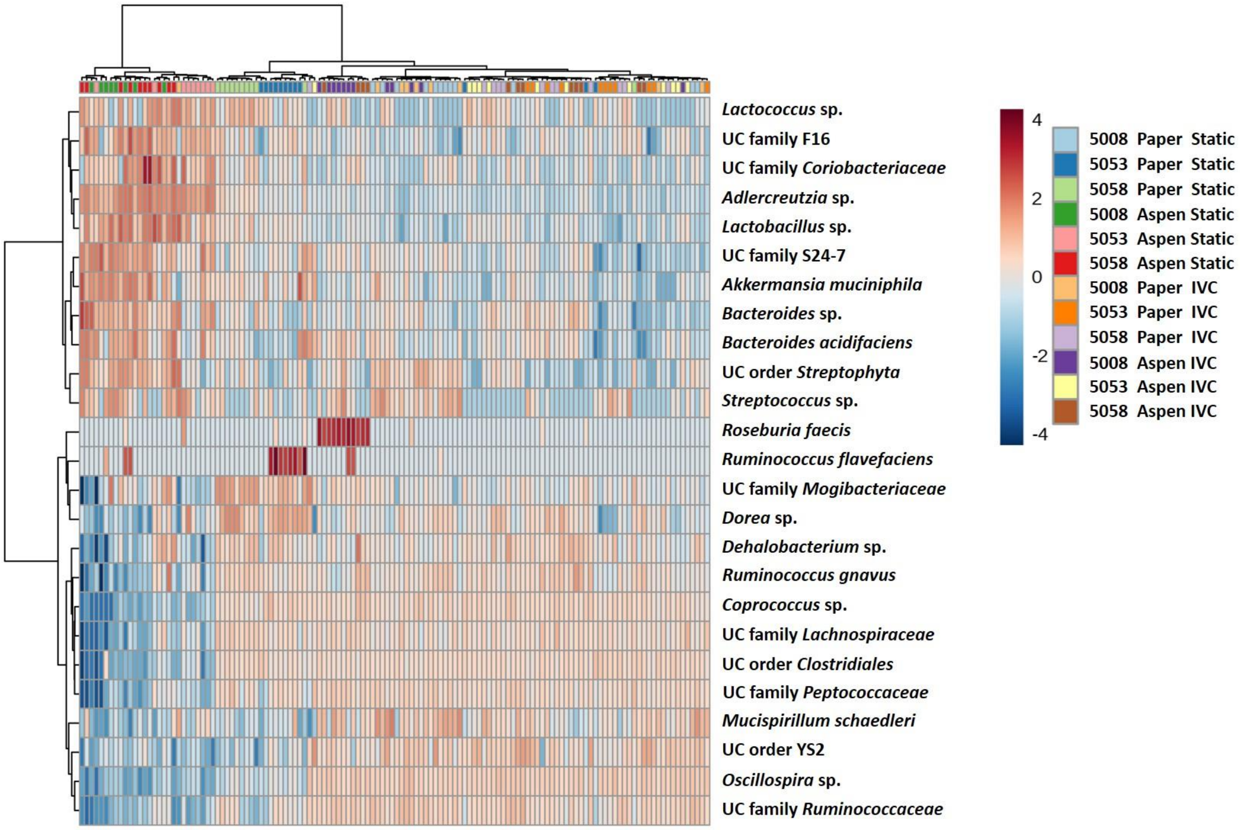

Supplementary Figure S3. Heatmap of log-fold relative abundance in endpoint cecal microbiota of top 25 differentially abundant operational taxonomic units (OTUs) as determined via analysis of variance, arranged via agglomerative hierarchical clustering of Euclidean distances between samples (x-axis) and OTUs (y-axis).

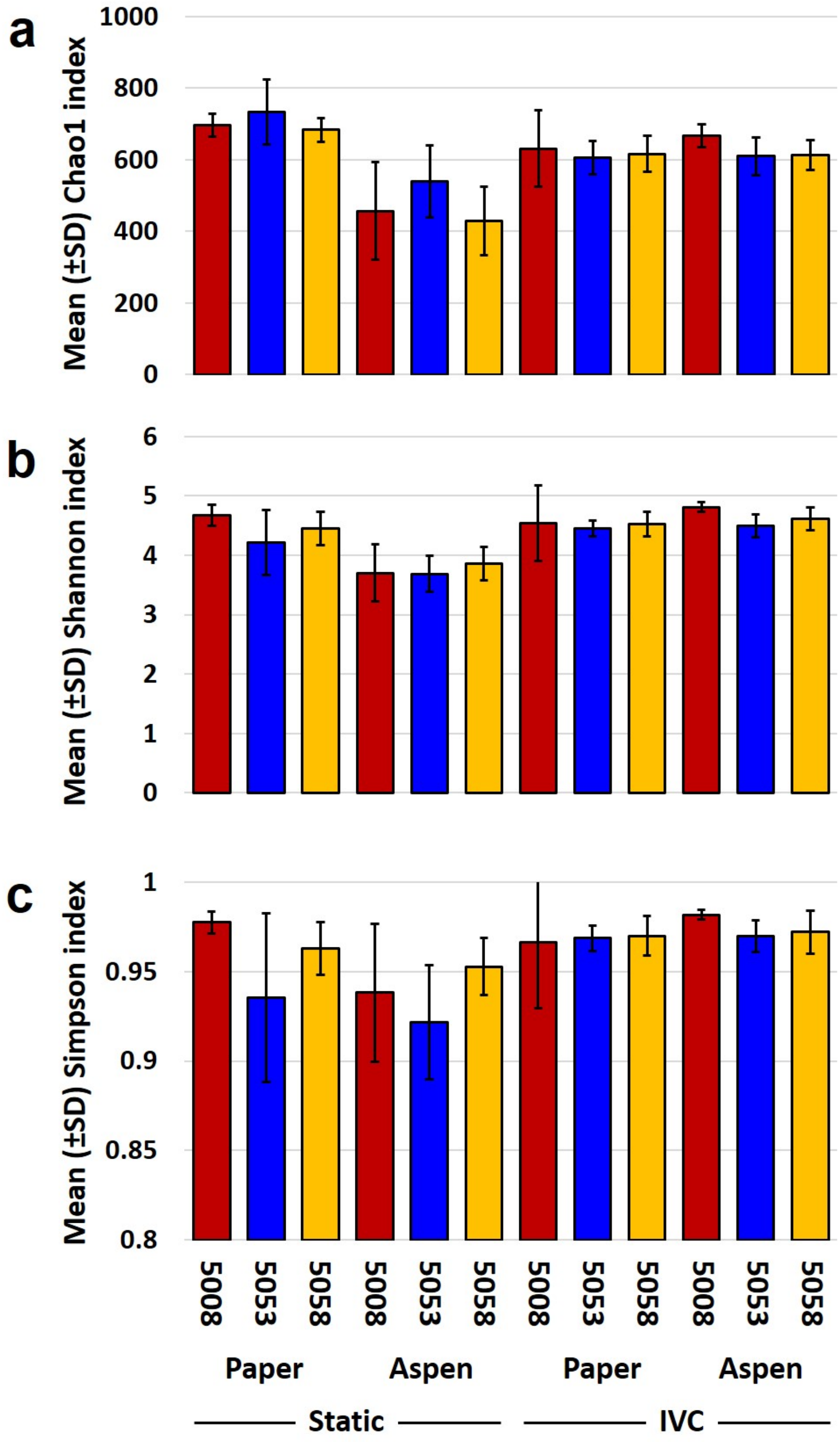

Supplementary Figure S4. Bar charts showing mean ± SD Chao1 (a), Shannon (b), and Simpson (c) diversity indices of cecal contents.

Supplementary Video S1. Rotating three-dimensional principal coordinates analysis of unweighted UniFrac distances with samples grouped according to their combination of housing and bedding. I = IVC, S = static, P = paper, and A = aspen.

**Caging**			**Static**	**Static**	**Static**	**Static**	**Static**	**Static**	**IVC**	**IVC**	**IVC**	**IVC**	**IVC**	**IVC**
	**Bedding**		**Paper**	**Paper**	**Paper**	**Aspen**	**Aspen**	**Aspen**	**Paper**	**Paper**	**Paper**	**Aspen**	**Aspen**	**Aspen**
		**Diet**	**5008**	**5053**	**5058**	**5008**	**5053**	**5058**	**5008**	**5053**	**5058**	**5008**	**5053**	**5058**
**Static**	**Paper**	**5008**		0.018	0.016	0.531	0.604	0.025	0.032	0.503	**<0.001**	0.228	0.008	0.112
**Static**	**Paper**	**5053**	3.37		0.251	0.025	0.025	0.065	0.047	0.026	**<0.001**	**<0.001**	**<0.001**	0.003
**Static**	**Paper**	**5058**	3.63	1.27		0.009	0.021	0.995	0.004	0.005	**<0.001**	0.002	**<0.001**	0.008
**Static**	**Aspen**	**5008**	0.74	3.33	4.52		0.547	0.013	0.343	0.844	**0.001**	0.213	0.103	0.345
**Static**	**Aspen**	**5053**	0.64	3.06	3.69	0.67		0.028	0.086	0.681	0.004	0.573	0.164	0.425
**Static**	**Aspen**	**5058**	3.18	2.54	0.01	4.46	3.38		0.003	0.009	**<0.001**	0.005	**<0.001**	0.016
**IVC**	**Paper**	**5008**	2.86	2.70	5.17	1.05	2.21	6.15		0.479	**<0.001**	0.008	0.009	0.078
**IVC**	**Paper**	**5053**	0.82	2.86	4.46	0.37	0.56	4.32	0.90		**0.001**	0.204	0.066	0.310
**IVC**	**Paper**	**5058**	14.18	24.44	25.48	11.05	7.23	22.20	16.95	11.15		0.006	0.011	0.011
**IVC**	**Aspen**	**5008**	1.40	6.89	6.80	1.42	0.63	5.75	4.56	1.50	7.16		0.179	0.453
**IVC**	**Aspen**	**5053**	4.07	9.53	11.86	2.16	1.66	10.40	5.02	2.29	4.73	1.57		0.306
**IVC**	**Aspen**	**5058**	2.02	4.86	4.82	1.03	0.88	4.09	2.50	1.14	5.89	0.80	1.15	

Supplementary Table S1. Results of PERMANOVA testing of Bray-Curtis distance matrices between the fecal microbiota of groups of mice housed under various combinations of caging (static microisolators versus individually ventilated cages (IVC)), bedding (compressed paper versus aspen chip), and dietary formulation, one week after arrival. Unadjusted p values shown in upper right; bold values withstood correction for multiple testing and maintained $p < 0.05$. Associated F values shown in lower left.

**Caging**			**Static**	**Static**	**Static**	**Static**	**Static**	**Static**	**IVC**	**IVC**	**IVC**	**IVC**	**IVC**	**IVC**
	**Bedding**		**Paper**	**Paper**	**Paper**	**Aspen**	**Aspen**	**Aspen**	**Paper**	**Paper**	**Paper**	**Aspen**	**Aspen**	**Aspen**
		**Diet**	**5008**	**5053**	**5058**	**5008**	**5053**	**5058**	**5008**	**5053**	**5058**	**5008**	**5053**	**5058**
**Static**	**Paper**	**5008**		0.010	**<0.001**	0.064	**<0.001**	**<0.001**	0.050	**<0.001**	0.002	0.005	**<0.001**	0.002
**Static**	**Paper**	**5053**	2.34		0.023	**<0.001**	0.086	0.002	0.001	**<0.001**	**<0.001**	**<0.001**	0.063	0.011
**Static**	**Paper**	**5058**	2.90	2.03		**<0.001**	0.049	0.060	0.090	0.003	0.007	0.255	0.030	0.888
**Static**	**Aspen**	**5008**	1.81	3.95	3.52		0.008	0.004	0.268	**<0.001**	0.003	0.013	0.002	0.018
**Static**	**Aspen**	**5053**	2.82	1.62	1.76	2.39		0.029	0.122	0.265	0.121	0.017	0.727	0.063
**Static**	**Aspen**	**5058**	2.84	2.50	1.72	2.65	1.86		0.010	**<0.001**	0.004	0.037	0.010	0.223
**IVC**	**Paper**	**5008**	1.79	3.01	1.56	1.25	1.45	2.12		0.019	0.003	0.409	0.006	0.094
**IVC**	**Paper**	**5053**	3.31	2.96	2.37	2.93	1.24	3.23	1.87		0.070	0.026	0.751	0.021
**IVC**	**Paper**	**5058**	2.73	2.90	2.36	2.78	1.51	2.42	2.39	1.60		0.005	0.971	0.049
**IVC**	**Aspen**	**5008**	2.54	3.24	1.26	2.30	2.01	1.84	1.06	1.81	2.15		0.072	0.407
**IVC**	**Aspen**	**5053**	2.70	1.73	1.91	2.96	0.76	2.12	2.25	0.74	0.32	1.64		0.067
**IVC**	**Aspen**	**5058**	2.70	2.18	0.49	2.24	1.68	1.31	1.62	2.06	1.85	1.05	1.72	

Supplementary Table S2. Results of PERMANOVA testing of Jaccard distance matrices between the fecal microbiota of groups of mice housed under various combinations of caging (static microisolators versus individually ventilated cages (IVC)), bedding (compressed paper versus aspen chip), and dietary formulation, one week after arrival. Unadjusted p values shown in upper right; bold values withstood correction for multiple testing and maintained $p < 0.05$. Associated F values shown in lower left.

**Caging**			**Static**	**Static**	**Static**	**Static**	**Static**	**Static**	**IVC**	**IVC**	**IVC**	**IVC**	**IVC**	**IVC**
	**Bedding**		**Paper**	**Paper**	**Paper**	**Aspen**	**Aspen**	**Aspen**	**Paper**	**Paper**	**Paper**	**Aspen**	**Aspen**	**Aspen**
		**Diet**	**5008**	**5053**	**5058**	**5008**	**5053**	**5058**	**5008**	**5053**	**5058**	**5008**	**5053**	**5058**
**Static**	**Paper**	**5008**		0.096	0.334	0.223	0.011	0.354	0.191	0.269	0.015	0.008	0.001	0.228
**Static**	**Paper**	**5053**	2.18		0.100	0.205	0.050	0.048	0.152	0.303	0.049	0.011	0.002	0.412
**Static**	**Paper**	**5058**	1.08	2.01		0.463	0.003	0.494	0.554	0.154	0.095	0.046	0.005	0.495
**Static**	**Aspen**	**5008**	1.41	1.46	0.81		0.010	0.513	0.451	0.159	0.092	0.059	0.003	0.907
**Static**	**Aspen**	**5053**	4.73	2.58	4.99	4.20		**<0.001**	0.011	0.364	0.007	0.001	0.002	0.010
**Static**	**Aspen**	**5058**	1.05	2.55	0.83	0.79	6.60		0.231	0.064	0.009	0.002	**0.001**	0.697
**IVC**	**Paper**	**5008**	1.67	1.78	0.52	0.73	4.68	1.42		0.139	0.491	0.375	0.037	0.505
**IVC**	**Paper**	**5053**	1.23	1.17	1.84	1.77	0.99	2.46	2.06		0.050	0.021	0.017	0.183
**IVC**	**Paper**	**5058**	4.76	2.69	2.19	2.21	4.60	4.48	0.76	3.01		0.943	0.322	0.096
**IVC**	**Aspen**	**5008**	6.03	3.95	3.01	2.68	7.33	6.40	0.94	4.33	0.25		0.102	0.042
**IVC**	**Aspen**	**5053**	10.41	6.50	6.58	7.18	7.70	12.53	3.64	5.21	1.14	2.07		0.003
**IVC**	**Aspen**	**5058**	1.42	0.98	0.77	0.27	4.09	0.55	0.71	1.66	2.22	2.91	7.37	

Supplementary Table S3. Results of PERMANOVA testing of Bray-Curtis distance matrices between the fecal microbiota of groups of mice housed under various combinations of caging (static microisolators versus individually ventilated cages (IVC)), bedding (compressed paper versus aspen chip), and dietary formulation for 13 weeks. Unadjusted *p* values shown in upper right; bold values withstood correction for multiple testing and maintained $p < 0.05$. Associated F values shown in lower left.

**Caging**			**Static**	**Static**	**Static**	**Static**	**Static**	**Static**	**IVC**	**IVC**	**IVC**	**IVC**	**IVC**	**IVC**
	**Bedding**		**Paper**	**Paper**	**Paper**	**Aspen**	**Aspen**	**Aspen**	**Paper**	**Paper**	**Paper**	**Aspen**	**Aspen**	**Aspen**
		**Diet**	**5008**	**5053**	**5058**	**5008**	**5053**	**5058**	**5008**	**5053**	**5058**	**5008**	**5053**	**5058**
**Static**	**Paper**	**5008**		0.002	0.012	0.034	**<0.001**	**<0.001**	0.028	**<0.001**	0.001	0.044	**<0.001**	0.032
**Static**	**Paper**	**5053**	2.98		0.368	0.003	0.034	0.006	0.006	**<0.001**	0.001	0.007	0.002	0.014
**Static**	**Paper**	**5058**	2.44	1.09		0.008	0.052	0.744	0.007	0.001	0.051	0.001	0.001	0.035
**Static**	**Aspen**	**5008**	2.12	3.49	2.93		**<0.001**	**<0.001**	0.017	**<0.001**	**0.001**	0.043	0.004	0.036
**Static**	**Aspen**	**5053**	4.78	2.15	1.85	5.71		0.001	0.001	**<0.001**	**<0.001**	**<0.001**	0.002	**<0.001**
**Static**	**Aspen**	**5058**	3.63	2.68	0.68	4.73	3.44		0.010	**<0.001**	**<0.001**	**<0.001**	**<0.001**	**<0.001**
**IVC**	**Paper**	**5008**	2.18	3.03	2.45	2.39	4.05	2.48		**0.001**	**<0.001**	0.011	**<0.001**	0.006
**IVC**	**Paper**	**5053**	4.14	4.02	4.00	5.24	5.06	6.96	4.68		**<0.001**	0.004	0.008	0.003
**IVC**	**Paper**	**5058**	3.70	3.72	2.05	3.47	5.38	4.67	4.57	3.69		0.010	0.036	0.112
**IVC**	**Aspen**	**5008**	2.08	2.73	3.16	2.17	4.05	5.25	2.85	3.93	2.64		0.034	0.247
**IVC**	**Aspen**	**5053**	3.65	3.39	3.08	3.08	2.92	5.51	3.94	2.39	2.16	2.30		0.019
**IVC**	**Aspen**	**5058**	2.08	2.43	1.92	2.02	4.04	3.20	2.91	2.98	1.62	1.31	2.32	

Supplementary Table S4. Results of PERMANOVA testing of Jaccard distance matrices between the fecal microbiota of groups of mice housed under various combinations of caging (static microisolators versus individually ventilated cages (IVC)), bedding (compressed paper versus aspen chip), and dietary formulation for 13 weeks. Unadjusted p values shown in upper right; bold values withstood correction for multiple testing and maintained $p < 0.05$. Associated F values shown in lower left.

Caging	Bedding	Diet	OTU count	Chao1	Shannon	Simpson
**Static**	**Paperchip**	**5008 (*n* = 12)**	48.5 ± 1.9	540 ± 48	4.34 ± 0.24	0.969 ± 0.009
**Static**	**Paperchip**	**5053 (*n* = 12)**	50.3 ± 4.2	591 ± 77	4.40 ± 0.32	0.964 ± 0.015
**Static**	**Paperchip**	**5058 (*n* = 7)**	48.4 ± 3.6	547 ± 52	4.38 ± 0.38	0.968 ± 0.015
**Static**	**Aspen**	**5008 (*n* = 10)**	46.6 ± 2.5	561 ± 81	4.47 ± 0.23	0.972 ± 0.007
**Static**	**Aspen**	**5053 (*n* = 12)**	50.1 ± 2.9	591 ± 74	4.30 ± 0.23	0.958 ± 0.012
**Static**	**Aspen**	**5058 (*n* = 12)**	48.6 ± 3.8	567 ± 60	4.42 ± 0.22	0.969 ± 0.008
**IVC**	**Paperchip**	**5008 (*n* = 6)**	47.3 ± 3.1	551 ± 92	4.58 ± 0.28	0.976 ± 0.004
**IVC**	**Paperchip**	**5053 (*n* = 7)**	44.1 ± 4.1	494 ± 98	4.02 ± 0.23	0.951 ± 0.012
**IVC**	**Paperchip**	**5058 (*n* = 10)**	45.8 ± 4.5	543 ± 66	4.29 ± 0.31	0.958 ± 0.016
**IVC**	**Aspen**	**5008 (*n* = 12)**	48.9 ± 4.5	603 ± 39	4.65 ± 0.12	0.976 ± 0.003
**IVC**	**Aspen**	**5053 (*n* = 11)**	46.6 ± 2.0	535 ± 46	4.28 ± 0.22	0.953 ± 0.015
**IVC**	**Aspen**	**5058 (*n* = 11)**	48.2 ± 3.5	543 ± 36	4.41 ± 0.15	0.970 ± 0.009

Supplementary Table S5. Mean ± standard deviation number of operational taxonomic units (OTU count), Chao1, Shannon, and Simpson diversity indices of the fecal microbiota of adult mice housed under various combinations of caging, bedding, and dietary formulations for 13 weeks. IVC = individually ventilated cages. Results of statistical testing for main effects and interactions provided in **Table 1** of the main text.

Caging			Static	Static	Static	Static	Static	Static	IVC	IVC	IVC	IVC	IVC	IVC
	Bedding		Paper	Paper	Paper	Aspen	Aspen	Aspen	Paper	Paper	Paper	Aspen	Aspen	Aspen
		Diet	5008	5053	5058	5008	5053	5058	5008	5053	5058	5008	5053	5058
**Static**	**Paper**	**5008**		0.090	0.920	0.260	0.326	0.004	0.095	0.098	0.032	0.017	0.015	0.018
**Static**	**Paper**	**5053**	2.35		0.122	0.110	0.042	**<0.001**	0.001	**0.001**	**<0.001**	**<0.001**	**<0.001**	**<0.001**
**Static**	**Paper**	**5058**	0.19	1.75		0.473	0.320	0.005	0.067	0.111	0.044	0.013	0.011	0.014
**Static**	**Aspen**	**5008**	1.31	1.96	0.76		0.031	**<0.001**	0.003	0.004	**0.001**	**<0.001**	**<0.001**	**<0.001**
**Static**	**Aspen**	**5053**	1.01	2.99	1.12	3.76		0.046	0.270	0.302	0.163	0.059	0.049	0.057
**Static**	**Aspen**	**5058**	6.56	17.95	7.34	20.55	3.01		0.774	0.362	0.968	0.487	0.055	0.213
**IVC**	**Paper**	**5008**	2.69	9.32	3.71	11.03	1.34	0.37		0.737	0.978	0.603	0.177	0.332
**IVC**	**Paper**	**5053**	2.55	8.59	2.64	9.03	1.15	0.92	0.26		0.634	0.438	0.172	0.394
**IVC**	**Paper**	**5058**	4.14	11.78	4.40	13.18	1.76	0.09	0.05	0.38		0.543	0.127	0.243
**IVC**	**Aspen**	**5008**	5.12	16.95	6.41	18.50	2.82	0.77	0.53	0.75	0.57		0.305	0.635
**IVC**	**Aspen**	**5053**	5.27	16.10	6.41	18.47	2.87	2.78	1.74	1.73	2.10	1.14		0.528
**IVC**	**Aspen**	**5058**	4.96	16.18	6.08	17.36	2.95	1.45	1.05	0.83	1.37	0.44	0.62	

Supplementary Table S6. Results of PERMANOVA testing of Bray-Curtis distances between the jejunal microbiota of groups of mice housed under various combinations of caging (static microisolators versus individually ventilated cages (IVC)), bedding (compressed paper versus aspen chip), and dietary formulation for 13 weeks. Unadjusted p values shown in upper right; bold values withstood correction for multiple testing with adjusted $p < 0.05$. Associated F values shown in lower left.

Caging			Static	Static	Static	Static	Static	Static	IVC	IVC	IVC	IVC	IVC	IVC
	Bedding		Paper	Paper	Paper	Aspen	Aspen	Aspen	Paper	Paper	Paper	Aspen	Aspen	Aspen
		Diet	5008	5053	5058	5008	5053	5058	5008	5053	5058	5008	5053	5058
**Static**	**Paper**	**5008**		0.034	0.260	0.180	0.045	0.004	0.021	**<0.001**	0.011	**<0.001**	**<0.001**	**<0.001**
**Static**	**Paper**	**5053**	1.86		0.243	0.054	0.115	0.018	0.008	**<0.001**	0.003	**<0.001**	**<0.001**	**<0.001**
**Static**	**Paper**	**5058**	1.14	1.20		0.158	0.800	0.075	0.038	0.016	0.175	0.002	**0.001**	**0.001**
**Static**	**Aspen**	**5008**	1.28	1.59	1.31		0.017	0.013	0.055	**<0.001**	**<0.001**	**<0.001**	**<0.001**	**<0.001**
**Static**	**Aspen**	**5053**	1.62	1.41	0.71	1.91		0.020	0.002	**<0.001**	0.004	**<0.001**	**<0.001**	**<0.001**
**Static**	**Aspen**	**5058**	2.89	1.94	1.56	1.86	2.03		0.052	**<0.001**	**0.001**	**<0.001**	**<0.001**	**<0.001**
**IVC**	**Paper**	**5008**	2.51	2.34	1.94	1.76	3.25	1.70		0.004	0.004	**0.001**	**<0.001**	**<0.001**
**IVC**	**Paper**	**5053**	3.13	3.45	2.11	3.81	2.89	4.13	3.27		0.176	0.201	0.058	0.021
**IVC**	**Paper**	**5058**	1.94	2.64	1.30	2.88	2.41	3.16	2.90	1.30		0.197	**<0.001**	0.004
**IVC**	**Aspen**	**5008**	2.93	3.82	2.60	3.72	3.25	4.99	4.05	1.23	1.23		0.010	0.017
**IVC**	**Aspen**	**5053**	4.44	5.05	3.33	6.06	3.65	6.99	5.14	1.65	2.82	1.99		0.001
**IVC**	**Aspen**	**5058**	3.94	4.42	2.73	4.57	3.76	4.69	3.88	1.81	2.07	1.76	2.30	

Supplementary Table S7. Results of PERMANOVA testing of Jaccard distance matrices between the jejunal microbiota of groups of mice housed under various combinations of caging (static microisolators versus individually ventilated cages (IVC)), bedding (compressed paper versus aspen chip), and dietary formulation for 13 weeks. Unadjusted p values shown in upper right; bold values withstood correction for multiple testing with adjusted $p < 0.05$. Associated F values shown in lower left.

**Caging**			**Static**	**Static**	**Static**	**Static**	**Static**	**Static**	**IVC**	**IVC**	**IVC**	**IVC**	**IVC**	**IVC**
	**Bedding**		**Paper**	**Paper**	**Paper**	**Aspen**	**Aspen**	**Aspen**	**Paper**	**Paper**	**Paper**	**Aspen**	**Aspen**	**Aspen**
		**Diet**	**5008**	**5053**	**5058**	**5008**	**5053**	**5058**	**5008**	**5053**	**5058**	**5008**	**5053**	**5058**
**Static**	**Paper**	**5008**		0.296	0.453	0.202	0.035	0.003	0.250	0.323	0.087	0.118	0.062	0.200
**Static**	**Paper**	**5053**	1.14		0.229	0.027	0.083	0.003	0.386	0.389	0.167	0.019	0.078	0.172
**Static**	**Paper**	**5058**	0.85	1.38		0.173	0.258	0.078	0.312	0.885	0.407	0.678	0.284	0.346
**Static**	**Aspen**	**5008**	1.50	3.84	1.61		0.004	0.003	0.029	0.133	0.041	0.102	0.058	0.039
**Static**	**Aspen**	**5053**	2.98	2.16	1.34	5.06		0.143	0.121	0.183	0.283	0.016	0.222	0.112
**Static**	**Aspen**	**5058**	4.34	3.79	2.02	5.55	1.70		0.031	0.106	0.477	0.017	0.172	0.078
**IVC**	**Paper**	**5008**	1.26	1.00	1.16	3.25	1.78	2.33		0.562	0.576	0.154	0.273	0.465
**IVC**	**Paper**	**5053**	1.09	0.96	0.27	1.97	1.58	1.95	0.73		0.818	0.398	0.620	0.317
**IVC**	**Paper**	**5058**	2.01	1.59	0.99	2.56	1.21	0.89	0.77	0.45		0.215	0.987	0.610
**IVC**	**Aspen**	**5008**	1.97	3.42	0.60	2.10	3.44	2.99	1.62	0.97	1.41		0.188	0.191
**IVC**	**Aspen**	**5053**	2.58	2.26	1.23	2.86	1.42	1.61	1.26	0.60	0.13	1.58		0.300
**IVC**	**Aspen**	**5058**	1.44	1.50	1.09	2.92	1.97	2.06	0.92	1.14	0.69	1.47	1.20	

Supplementary Table S8. Results of PERMANOVA testing of Bray-Curtis distance matrices between the ileal microbiota of groups of mice housed under various combinations of caging (static microisolators versus individually ventilated cages (IVC)), bedding (compressed paper versus aspen chip), and dietary formulation for 13 weeks. Unadjusted p values shown in upper right; bold values withstood correction for multiple testing with adjusted $p < 0.05$. Associated F values shown in lower left.

**Caging**			**Static**	**Static**	**Static**	**Static**	**Static**	**Static**	**IVC**	**IVC**	**IVC**	**IVC**	**IVC**	**IVC**
	**Bedding**		**Paper**	**Paper**	**Paper**	**Aspen**	**Aspen**	**Aspen**	**Paper**	**Paper**	**Paper**	**Aspen**	**Aspen**	**Aspen**
		**Diet**	**5008**	**5053**	**5058**	**5008**	**5053**	**5058**	**5008**	**5053**	**5058**	**5008**	**5053**	**5058**
**Static**	**Paper**	**5008**		0.031	0.021	0.020	0.006	**0.001**	0.002	**<0.001**	**<0.001**	0.026	0.002	**<0.001**
**Static**	**Paper**	**5053**	1.82		0.336	0.078	0.133	0.018	0.005	**<0.001**	0.002	0.051	0.037	0.001
**Static**	**Paper**	**5058**	1.82	1.08		0.003	0.119	0.136	**<0.001**	**<0.001**	0.003	0.009	0.033	0.047
**Static**	**Aspen**	**5008**	1.82	1.57	2.14		**<0.001**	0.001	0.003	**<0.001**	**<0.001**	0.070	0.091	**0.001**
**Static**	**Aspen**	**5053**	2.22	1.40	1.35	2.65		0.020	**0.001**	**<0.001**	**<0.001**	0.009	**0.001**	**0.001**
**Static**	**Aspen**	**5058**	2.25	1.88	1.29	2.29	1.79		**<0.001**	**<0.001**	0.001	0.004	**0.001**	0.015
**IVC**	**Paper**	**5008**	1.97	2.23	2.51	1.96	2.81	2.37		**<0.001**	0.001	0.032	0.013	0.002
**IVC**	**Paper**	**5053**	3.33	3.61	3.17	3.69	3.64	2.70	2.31		0.001	**<0.001**	**<0.001**	**<0.001**
**IVC**	**Paper**	**5058**	2.90	2.69	2.06	2.41	3.28	2.08	2.10	2.39		0.008	0.063	0.533
**IVC**	**Aspen**	**5008**	1.97	1.81	2.16	1.59	2.39	2.30	1.70	3.35	2.28		0.095	0.010
**IVC**	**Aspen**	**5053**	2.03	1.62	1.53	1.39	2.23	2.12	1.63	2.30	1.40	1.41		0.114
**IVC**	**Aspen**	**5058**	2.48	2.51	1.55	2.35	2.63	1.72	2.10	2.94	0.93	2.11	1.33	

Supplementary Table S9. Results of PERMANOVA testing of Jaccard distance matrices between the ileal microbiota of groups of mice housed under various combinations of caging (static microisolators versus individually ventilated cages (IVC)), bedding (compressed paper versus aspen chip), and dietary formulation for 13 weeks. Unadjusted p values shown in upper right; bold values withstood correction for multiple testing with adjusted $p < 0.05$. Associated F values shown in lower left.

**Caging**			**Static**	**Static**	**Static**	**Static**	**Static**	**Static**	**IVC**	**IVC**	**IVC**	**IVC**	**IVC**	**IVC**
	**Bedding**		**Paper**	**Paper**	**Paper**	**Aspen**	**Aspen**	**Aspen**	**Paper**	**Paper**	**Paper**	**Aspen**	**Aspen**	**Aspen**
		**Diet**	**5008**	**5053**	**5058**	**5008**	**5053**	**5058**	**5008**	**5053**	**5058**	**5008**	**5053**	**5058**
**Static**	**Paper**	**5008**		**<0.001**	**<0.001**	**<0.001**	**<0.001**	**<0.001**	0.551	**<0.001**	0.028	0.099	0.013	0.631
**Static**	**Paper**	**5053**	8.30		0.398	**<0.001**	**<0.001**	**<0.001**	0.030	0.016	0.002	**0.003**	0.008	**0.002**
**Static**	**Paper**	**5058**	10.64	0.98		**<0.001**	**<0.001**	**<0.001**	0.005	**<0.001**	**<0.001**	**<0.001**	**<0.001**	**<0.001**
**Static**	**Aspen**	**5008**	50.87	27.89	26.90		0.039	0.225	**<0.001**	**<0.001**	**<0.001**	**<0.001**	**<0.001**	**<0.001**
**Static**	**Aspen**	**5053**	35.51	17.73	17.26	3.71		0.020	**<0.001**	**<0.001**	**<0.001**	**<0.001**	**<0.001**	**<0.001**
**Static**	**Aspen**	**5058**	56.71	33.12	32.02	1.38	4.20		**<0.001**	**<0.001**	**<0.001**	**<0.001**	**<0.001**	**<0.001**
**IVC**	**Paper**	**5008**	0.87	2.77	3.22	19.04	13.9	22.41		0.308	0.226	0.591	0.268	0.276
**IVC**	**Paper**	**5053**	5.84	4.14	5.91	39.18	25.43	44.58	1.18		0.102	0.010	0.168	0.022
**IVC**	**Paper**	**5058**	2.31	5.41	6.01	38.94	28.57	45.16	1.36	1.85		0.193	0.589	0.636
**IVC**	**Aspen**	**5008**	1.82	7.28	8.35	41.24	29.66	46.84	0.77	3.69	1.48		0.015	0.176
**IVC**	**Aspen**	**5053**	3.74	4.66	6.06	45.89	31.1	51.81	1.26	1.62	0.77	3.04		0.149
**IVC**	**Aspen**	**5058**	2.09	6.94	7.55	43.35	31.65	49.27	1.20	3.17	0.69	1.55	1.63	

Supplementary Table S10. Results of PERMANOVA testing of Bray-Curtis distance matrices between the cecal microbiota of groups of mice housed under various combinations of caging (static microisolators versus individually ventilated cages (IVC)), bedding (compressed paper versus aspen chip), and dietary formulation for 13 weeks. Unadjusted p values shown in upper right; bold values withstood correction for multiple testing with adjusted $p < 0.05$. Associated F values shown in lower left.

**Caging**			**Static**	**Static**	**Static**	**Static**	**Static**	**Static**	**IVC**	**IVC**	**IVC**	**IVC**	**IVC**	**IVC**
	**Bedding**		**Paper**	**Paper**	**Paper**	**Aspen**	**Aspen**	**Aspen**	**Paper**	**Paper**	**Paper**	**Aspen**	**Aspen**	**Aspen**
		**Diet**	**5008**	**5053**	**5058**	**5008**	**5053**	**5058**	**5008**	**5053**	**5058**	**5008**	**5053**	**5058**
**Static**	**Paper**	**5008**		**<0.001**	**<0.001**	**<0.001**	**<0.001**	**<0.001**	**<0.001**	**<0.001**	**<0.001**	**<0.001**	**<0.001**	**<0.001**
**Static**	**Paper**	**5053**	3.92		0.063	0.003	0.003	**<0.001**	0.001	**<0.001**	**<0.001**	**<0.001**	**<0.001**	**<0.001**
**Static**	**Paper**	**5058**	6.21	1.94		0.002	0.032	0.002	**<0.001**	**<0.001**	**<0.001**	**<0.001**	**<0.001**	**<0.001**
**Static**	**Aspen**	**5008**	5.21	3.20	2.92		0.025	0.246	0.007	**<0.001**	**<0.001**	**<0.001**	**<0.001**	**<0.001**
**Static**	**Aspen**	**5053**	6.56	2.86	2.21	2.17		0.011	**<0.001**	**<0.001**	**<0.001**	**<0.001**	0.002	**<0.001**
**Static**	**Aspen**	**5058**	5.24	4.95	3.59	1.30	2.38		0.002	**<0.001**	**<0.001**	**<0.001**	**<0.001**	0.002
**IVC**	**Paper**	**5008**	3.53	4.09	4.91	2.57	5.07	3.41		**0.001**	0.080	0.097	0.016	**<0.001**
**IVC**	**Paper**	**5053**	6.35	6.64	7.90	4.58	3.83	4.41	3.86		**<0.001**	**<0.001**	0.175	0.022
**IVC**	**Paper**	**5058**	5.58	3.85	4.15	5.21	5.81	5.15	1.81	6.11		0.003	0.032	0.068
**IVC**	**Aspen**	**5008**	5.06	4.05	5.86	4.36	5.06	4.71	1.71	4.68	3.00		0.019	0.005
**IVC**	**Aspen**	**5053**	4.22	4.32	4.51	4.21	3.10	3.72	2.61	1.43	2.23	2.62		0.029
**IVC**	**Aspen**	**5058**	5.32	5.21	4.87	3.35	5.46	3.06	2.40	4.69	1.89	3.27	2.40	

Supplementary Table S11. Results of PERMANOVA testing of Jaccard distance matrices between the cecal microbiota of groups of mice housed under various combinations of caging (static microisolators versus individually ventilated cages (IVC)), bedding (compressed paper versus aspen chip), and dietary formulation for 13 weeks. Unadjusted p values shown in upper right; bold values withstood correction for multiple testing with adjusted $p < 0.05$. Associated F values shown in lower left.

Caging	Bedding	Diet	OTU count	Chao1	Shannon	Simpson
**Static**	**Paperchip**	**5008 (*n* = 12)**	48.2 ± 1.8	696 ± 32	4.67 ± 0.17	0.978 ± 0.006
**Static**	**Paperchip**	**5053 (*n* = 12)**	49.4 ± 4.5	734 ± 90	4.21 ± 0.54	0.936 ± 0.047
**Static**	**Paperchip**	**5058 (*n* = 11)**	48.7 ± 2.1	783 ± 34	4.45 ± 0.28	0.963 ± 0.015
**Static**	**Aspen**	**5008 (*n* = 8)**	43.9 ± 5.6	457 ± 137	3.70 ± 0.48	0.938 ± 0.039
**Static**	**Aspen**	**5053 (*n* = 9)**	46.4 ± 3.0	540 ± 101	3.69 ± 0.30	0.922 ± 0.032
**Static**	**Aspen**	**5058 (*n* = 10)**	42.8 ± 5.4	429 ± 96	3.86 ± 0.28	0.953 ± 0.016
**IVC**	**Paperchip**	**5008 (*n* = 9)**	44.3 ± 3.8	631 ± 108	4.54 ± 0.64	0.967 ± 0.037
**IVC**	**Paperchip**	**5053 (*n* = 11)**	43.3 ± 3.3	605 ± 47	4.45 ± 0.14	0.969 ± 0.007
**IVC**	**Paperchip**	**5058 (*n* = 12)**	45.6 ± 1.8	616 ± 50	4.52 ± 0.20	0.970 ± 0.011
**IVC**	**Aspen**	**5008 (*n* = 12)**	46.8 ± 2.6	667 ± 32	4.81 ± 0.08	0.982 ± 0.003
**IVC**	**Aspen**	**5053 (*n* = 12)**	43.4 ± 1.8	610 ± 54	4.49 ± 0.19	0.970 ± 0.009
**IVC**	**Aspen**	**5058 (*n* = 12)**	44.0 ± 4.5	614 ± 42	4.62 ± 0.19	0.972 ± 0.012

Supplementary Table S12. Mean ± standard deviation number of operational taxonomic units (OTU count), Chao1, Shannon, and Simpson diversity indices of the cecal microbiota of adult mice housed under various combinations of caging, bedding, and dietary formulations for 13 weeks. IVC = individually ventilated cages. Results of statistical testing for main effects and interactions provided in **Table 2** of the main text.
