# Supplementary figures and images for: The influence of caging, bedding, and diet on the composition of the microbiota in different regions of the mouse gut
Source: Sci Rep. 2018 Mar 6;8:4065. doi: 10.1038/s41598-018-21986-7 (PMC5840362; doi:10.1038/s41598-018-21986-7)

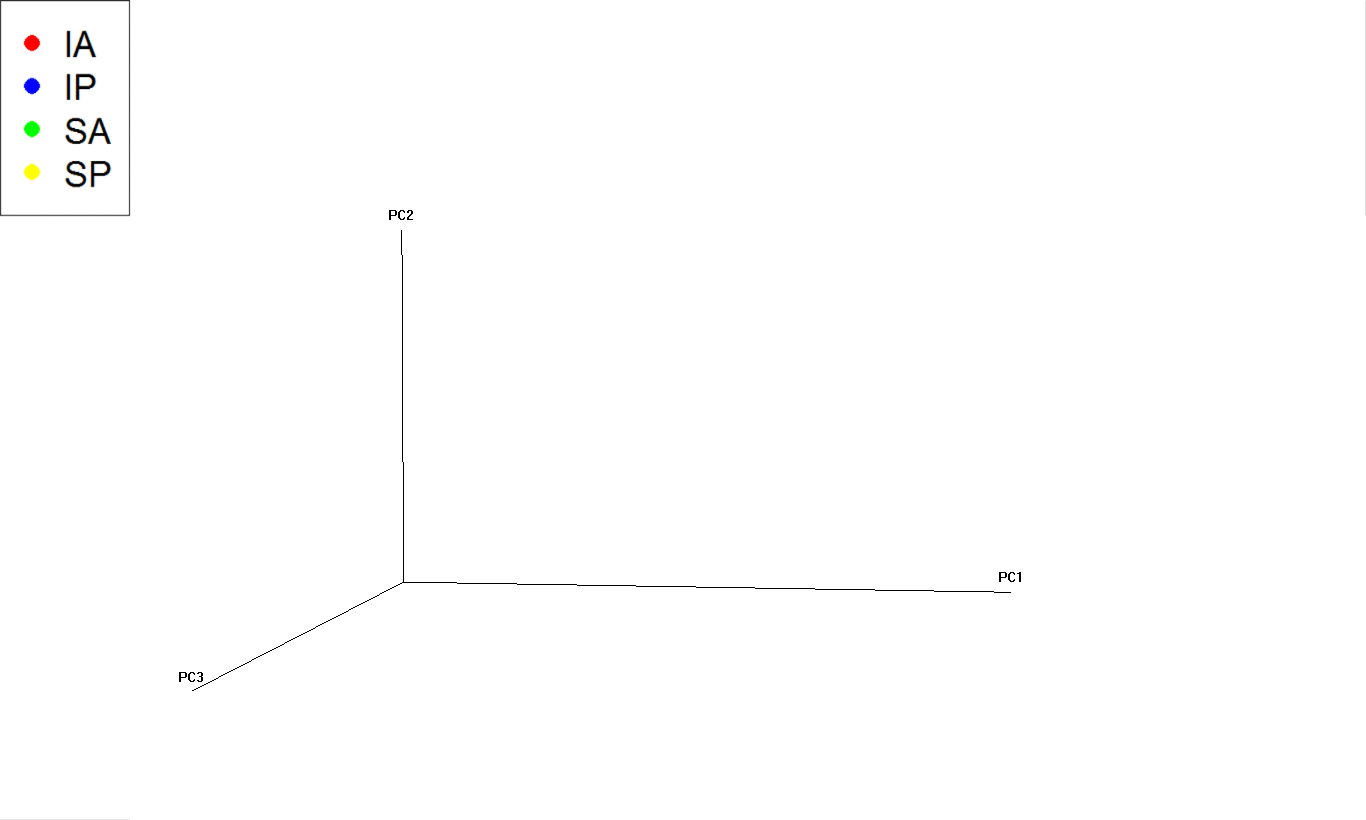

Supplement: Supplementary file 2 — Supplementary Video S1. [file 41598_2018_21986_MOESM2_ESM.gif]
